# The Role of Mutated Calreticulin in the Pathogenesis of *BCR-ABL1*-Negative Myeloproliferative Neoplasms

**DOI:** 10.3390/ijms25189873

**Published:** 2024-09-12

**Authors:** Roberta Vadeikienė, Baltramiejus Jakštys, Danguolė Laukaitienė, Saulius Šatkauskas, Elona Juozaitytė, Rasa Ugenskienė

**Affiliations:** 1 Oncology Research Laboratory, Institute of Oncology, Lithuanian University of Health Sciences, LT-50161 Kaunas, Lithuania; 2 Research on Delivery of Medicine and Genes Cluster, Faculty of Natural Sciences, Vytautas Magnus University, LT-44001 Kaunas, Lithuaniasaulius.satkauskas@vdu.lt (S.Š.); 3 Institute of Oncology, Lithuanian University of Health Sciences, LT-50161 Kaunas, Lithuania; 4 Department of Genetics and Molecular Medicine, Lithuanian University of Health Sciences, LT-50161 Kaunas, Lithuania

**Keywords:** calreticulin, myeloproliferative neoplasms, JAK/STAT, PI3K/Akt/mTOR, Hedgehog, oxidative stress, DNA damage, apoptosis, cell cycle

## Abstract

Myeloproliferative neoplasms (MPNs) are characterized by increased proliferation of myeloid lineages in the bone marrow. Calreticulin (*CALR*) 52 bp deletion and *CALR* 5 bp insertion have been identified in essential thrombocythemia (ET) and primary myelofibrosis (PMF). There is not much data on the crosstalk between mutated *CALR* and MPN-related signaling pathways, such as JAK/STAT, PI3K/Akt/mTOR, and Hedgehog. Calreticulin, a multifunctional protein, takes part in many cellular processes. Nevertheless, there is little data on how mutated *CALR* affects the oxidative stress response and oxidative stress-induced DNA damage, apoptosis, and cell cycle progression. We aimed to investigate the role of the *CALR* 52 bp deletion and 5 bp insertion in the pathogenesis of MPN, including signaling pathway activation and functional analysis in *CALR*-mutated cells. Our data indicate that the JAK/STAT and PI3K/Akt/mTOR pathways are activated in *CALR*-mutated cells, and this activation does not necessarily depend on the CALR and MPL interaction. Moreover, it was found that *CALR* mutations impair calreticulin function, leading to reduced responses to oxidative stress and DNA damage. It was revealed that the accumulation of G2/M-*CALR*-mutated cells indicates that oxidative stress-induced DNA damage is difficult to repair. Taken together, this study contributes to a deeper understanding of the specific molecular mechanisms underlying *CALR*-mutated MPNs.

## 1. Introduction

Myeloproliferative neoplasms (MPNs) are a heterogeneous group of hematologic malignancies resulting from mutant hematopoietic stem/progenitor cells. According to the World Health Organization (WHO), classical Philadelphia chromosome-negative (*BCR-ABL1*-negative) MPNs are classified into primary myelofibrosis (PMF), essential thrombocythemia (ET), and polycythemia vera (PV). PMF is characterized by excessive bone marrow scarring and fibrosis, ET by excessive platelet production, and PV by excessive red cell production [1,2]. Clinically, *BCR-ABL1*-negative MPNs share the features of bone marrow hypercellularity and an increased risk of thrombosis or hemorrhage; incurable MPNs can lead to acute leukemia [3,4]. In addition to being classified as a myeloproliferative neoplasm, chronic myeloid leukemia (CML) is distinguished by being *BCR-ABL1* positive [1,2].

Each MPN has unique clinical features with a unifying theme of somatic acquisition of a mutation in either *JAK2* (Janus kinase 2), *MPL* (thrombopoietin receptor), or *CALR* (calreticulin) in hematopoietic stem cells. Carriers of the mentioned genetic mutations have a higher risk of developing PMF, PV, and ET; however, the exact cause of these diseases remains unknown. Environmental and behavioral risk factors, e.g., exposure to ionizing radiation, carcinogenic and immunotoxic agents, as well as a history of smoking, have also been associated with an increased risk of *BCR-ABL1* negative MPNs [5,6]. While analyzing the genetic basis of MPNs, it was determined that more than 90% of PV patients carry the *JAK2* p.V617F mutation; however, around 2% of PV patients do not have *JAK2* p.V617F, but insertions and deletions in exon 12 [7,8,9]. About 90% of ET cases represent a molecular clonal marker: *JAK2* p.V617F (50–60%), *MPL* p.W515L/K (3–15%) mutations, and *CALR* 52 bp deletion or 5 bp insertion (20–25%) [10,11,12]. Patients with PMF harbor one of three somatic mutations: *JAK2* p.V617F (up to 60%), *MPL* p.W515L/K (up to 15%), and *CALR* 52 bp deletion or 5 bp insertion (up to 20%) [13]. Recently, mutations in epigenetic regulators and RNA splicing genes, e.g., *TET2*, *SRSF2*, *IDH2*, and *ASXL1*, have also been found in patients with MPNs. However, the mentioned mutations do not have any diagnostic impact in most cases because of their low frequency and specificity [14]. In the last decade, advances in understanding the molecular mechanisms underlying excessive myeloproliferation have been achieved, and novel targeted therapies for MPNs have been developed, e.g., ruxolitinib, a specific JAK1/2 inhibitor, is the first agent approved to treat high-risk patients with PV and PMF [15]. However, ruxolitinib has limited use in that most patients with MPN progress or become intolerant within 2–3 years [16]. Considering the heterogeneous genomic landscape, additional mutations in MPN could represent diagnostic and prognostic value, leading to the risk of progression and informing decisions on therapeutic management. Therefore, there is still a need for novel MPN therapeutic options rationally designed based on recent molecular mechanistic insights.

The first findings of somatic mutations in the calreticulin gene that drive myeloproliferation were reported in 2013. *CALR* mutations are found in a significant proportion of patients with PMF and ET and, as mentioned before, are the second most common gene alterations that drive myeloproliferation [17]. Changes in *CALR* are mutually exclusive with mutations in *JAK2* and *MPL* genes [10,18]. Although the coexistence of the two driver mutations has been reported, these are rare events, and their clinical-prognostic implications are under investigation [19]. More than 50 *CALR* mutations have been identified, and all known variants cause a +1 bp frameshift in the reading frame of exon 9 and generate a novel terminal amino acid sequence common to all mutant calreticulin proteins [20]. A 52 bp deletion (type 1: c.1092_1143del) and a 5 bp insertion (type 2: c.1154_1155insTTGTC) are accepted as the most frequent forms of mutated *CALR* found in PMF and ET patients. It has been determined that the aforementioned phenotypic driver mutations in MPNs have an impact on genes that are directly involved in cytokine signaling (e.g., *JAK2*, *MPL*, *BCR-ABL1*) [21,22,23]. *CALR* instead encodes an endoplasmic reticulum (ER) chaperone that functions in calcium ion homeostasis, cell adhesion, antigen presentation, danger signaling, and cell death, and assists glycoproteins in obtaining their mature structure [24,25]. A systematic investigation of *CALR* mutations revealed that the loss of the C-terminal KDEL motif leads to reduced endoplasmic reticulum retention of mutated calreticulin and impairs the aforementioned multiple native calreticulin functions [26]. From a clinical viewpoint, it was observed that the presence of *CALR* mutations is related to a clinically distinctive and good prognosis of ET and PMF, compared to *JAK2*-mutated ones. However, it is important to note that a worse MPN course, i.e., increased risk of transformation of ET to secondary myelofibrosis, splenomegaly, aspirin-induced hemorrhage, and worsened survival, was also observed in patients harboring a *CALR* 52 bp deletion and 5 bp insertion [10,17,18,27,28,29,30,31,32]. In addition, it was observed that *CALR* gene mutations appear early in ET and PMF; hence, this could be used as a transformation marker [33]. Active investigations on how mutated *CALR* induces cell transformation are being carried out, yet there are no conclusive data about mutated *CALR’s* contribution to MPN pathogenesis. Therefore, the need arises for a deeper understanding of the molecular mechanisms underlying the characteristics of *CALR*-mutated MPNs. The identification of aberrant mechanisms of mutated calreticulin and its role in MPN etiopathogenesis would serve to develop novel molecular targeted therapy approaches for MPNs in the future.

The analysis of the molecular mechanism of mutated calreticulin is complicated because a commercial cell line carrying a *CALR* 52 bp deletion or 5 bp insertion has not yet been established. To our knowledge, the only commercial cell line to harbor *CALR* mutation is MARIMO. However, this cell line is characterized by a 61 bp deletion in exon 9 [34,35,36,37]. The function of *CALR* 61 bp deletion in the MARIMO cell line remains unclear, and the authors question the use of these cells as a model for the malign function of *CALR* mutants. The way out of the problem of lacking a commercial cell line is to initiate common *CALR* mutations in relatively easy-to-transfect cells, such as HEK293, 32D, or Ba/F3 cells [10,38,39]. Recently, several studies have been conducted with human suspension cell lines that were also transfected, and an analysis related to *CALR*’s mutation role in MPNs was performed [40,41,42]. Nevertheless, the use of suspension cell lines is complicated because these cells are difficult to transfect using conventional transfection methods, such as liposomal or calcium phosphate methods [43]. It is difficult to find an appropriate cellular model reflecting the genetic basis and molecular profiling of MPNs, so a few research groups have analyzed calreticulin’s role in these diseases using circulating CD34^+^ cells or cells cultured from blood or bone marrow samples of patients with MPN [38,44,45,46,47]. It can be added that the amount of research related to patient cell samples is limited because not all research institutions have access to patient blood and bone marrow samples. Furthermore, the preparation and cultivation of cells derived from patients is complicated, cost-effective, and has technical limitations compared to commercial cell lines. Considering that originally, mutationally, and adhesively different cellular models have been used in previous studies, mutated *CALR*-related research data remains conflicting.

The Janus kinase/signal transducer and activator of the transcription (JAK/STAT) pathway is one of the central junctions of interaction in cell functions, e.g., hemopoiesis, immune cell development, tissue repair, stem cell maintenance, apoptosis, and adipogenesis [48]. It is known that JAK/STAT is a highly conserved pathway of signal transduction, and dysregulation of this signaling is associated with various diseases, including MPNs, Hodgkin lymphoma, and hepatocellular carcinoma [48,49,50]. As mentioned before, MPN-related molecular abnormalities play a crucial role in the constitutive activation of JAK/STAT signaling. A few in vitro studies have also analyzed the impact of the mutated *CALR* on the activation of this signaling pathway [10,34,51,52,53]. Increased activation of JAK/STAT signaling has been reported, but it is important to note that this activation is unique due to the capability of the novel C-terminus of the *CALR* mutant to bind directly to MPL [10,51,52,54]. This statement was clarified in vivo, where *c-mpl*-deficient mice were protected from MPN disease with the *CALR* phenotype [55,56]. Moreover, *CALR*-mutated PMF patients responded to JAK2 inhibition, and control of the symptoms and quality of life were improved, which also shows CALR involvement in JAK/STAT signaling [55,57,58]. Contrary to the mentioned results, other studies’ molecular analysis showed no association between mutated calreticulin and JAK/STAT but represented the increased activity of ERK1/2, MAPK, and PI3K/Akt/mTOR [36,37,53,56,59,60]. Furthermore, it has recently been suggested that the consequences of *CALR* mutations on megakaryopoiesis are likely to be mediated not only by MAPK but also by STAT5 signaling, which is accompanied by MPL-dependent activation [34]. Based on the conflicting results of the mentioned studies, there is a need for further research and elucidation of the effect of the mutated *CALR* on JAK/STAT signaling pathway activation.

The PI3K/Akt/mTOR (phosphatidylinositol 3-kinase/Protein kinase B/mammalian target of rapamycin) signaling pathway is involved in most cellular processes, e.g., cell proliferation, adhesion, cell death, migration, and invasion. It was noticed that PIK3/Akt/mTOR is commonly activated in human cancers [61,62,63]. The constitutive activation of the PI3K/Akt/mTOR signaling pathway was determined to be central to MPN pathogenesis. In addition, it was suggested that the PI3K/Akt/mTOR cascade regulates cell growth and proliferation signals downstream of the JAK/STAT signaling pathway [60]. Preclinical studies have shown that this signaling pathway inhibitor alone and in combination with JAK/STAT inhibitors reduced proliferation and induced apoptosis in cells carrying *JAK2* or *MPL* mutations [60,64,65,66,67]. However, there is little data that represent mutated calreticulin’s impact on the PI3K/Akt/mTOR signaling pathway. Independent research groups have determined that *CALR* mutants induce the phosphorylation of JAK2, STAT1, STAT3, and STAT5, while PIK3/Akt/mTOR and MAPK signaling systems are weakly activated [51,55]. However, it is important to note that this kind of signaling activation is also dependent on human *MPL* expression. Kollman et al. (2017) revealed that *CALR*-mutant murine 32D cell line without exogenous MPL showed increased Akt phosphorylation [36]. Considering the controversial and limited results of the mentioned studies, it is necessary to fully elucidate the mechanism that determines crosstalk between mutated *CALR* and the PI3K/Akt/mTOR signaling pathway.

The Hedgehog (Hh) signaling pathway is a highly conserved pathway of signal transduction from the cell membrane to the nucleus. Hh signaling plays an important role in normal embryonic growth and the development of many tissues and organs. Lately, it has been shown that the Hh pathway plays a role in carcinogenesis in different tumors, as well as in the pathogenesis of hematologic malignancies [68,69,70,71,72,73]. However, there is little data on the association between the Hh signaling pathway and MPNs [74,75,76]. Bhagwat and coauthors [74] reported that the expression of *PTCH1* and *GLI1* was increased up to 100-fold in granulocytes derived from patients with MPNs compared with control granulocytes. In addition, it was found that components of the Hh pathway cooperate with other signaling pathways to produce the biological phenotype of PMF [76]. Contrary to these results, it was found that in the *C. elegans* model with initiated *CALR* 52 deletion and *CALR* 5 bp insertion, most of the Hedgehog signaling pathway targets were downregulated [77]. Moreover, these results are reinforced by the Lucijanic et al. (2020) study, in which *GLI1* expression was analyzed in patients with myelofibrosis and healthy controls. The findings of the mentioned study imply that the upregulation of *GLI1* does not appear to be a characteristic of myelofibrosis etiopathogenesis [78]. Therefore, due to inconclusive data, it is necessary to expand the current experiments analyzing mutant calreticulin and Hedgehog signaling pathway association.

Several studies have revealed the importance of reactive oxygen species (ROS) in MPNs: MPN patients demonstrated increased levels of ROS [79], and cellular models exhibited the production of ROS in vitro [80]. In addition, separate research groups have reported that *JAK2*, *MPL*, and *CALR* variants are associated with other DNA alterations leading to an inflammatory state characterized by excessive production of ROS [81,82]. Apart from the activation of cell signaling pathways, calreticulin, a multifunctional protein, is involved in many cellular processes. The impact of *CALR* 52 bp deletion and 5 bp insertion in the pathogenesis of MPNs has been partly clarified, but there is little data that describes the effects of the mentioned mutations on the physiological functions of calreticulin. Recently, it has been shown that cells overexpressing *CALR* demonstrate increased sensitivity to ER and oxidative stress [40,83]. To our knowledge, only a few studies have analyzed mutated calreticulin’s impact on oxidative stress in a cell culture model [40,41]. It was determined that cells expressing mutated *CALR* showed increased ROS intracellular levels and increased levels of DNA damage upon oxidative stress exposure using Melittin and Miltirone. Additionally, CALR mutants were characterized by a decreased ability to reduce intracellular ROS levels and repair oxidative DNA damage. It is known that calreticulin is involved in various biological processes, including cell death. However, only Genovese with colleagues [41] data showed an increase in oxidative stress-induced apoptosis levels in cells carrying *CALR* mutations. Taken together, functional analysis for a deeper understanding of the *CALR* 52 bp deletion and 5 bp insertion on native calreticulin functions is essential.

To sum up, the pathogenesis of MPNs is very complex, and dysregulated signaling pathways and their crosstalk with mutated calreticulin may offer additional therapeutic targets. Therefore, there is a need for further research and elucidation of the mechanism underlying the crosstalk between mutated *CALR* and cell signaling pathways, JAK/STAT, PI3K/Akt/mTOR, and Hedgehog, which are important in MPN pathogenesis. In this study, we explored the potent effect of targeting JAK/STAT, PI3K/Akt/mTOR, and Hedgehog signaling pathways with specific inhibitors in vitro and subsequently carried out target gene expression and protein level analysis in cells carrying the *CALR* 52 bp deletion and 5 bp insertion, as well as in cells with *JAK2* p.V617F and *JAK2*/*CALR* wild-type. This research is unique because most studies analyze mutant *CALR* when *MPL* expression is present, but we performed experiments in a cell culture model lacking *MPL* expression. In this complex study, we also explored how *CALR* 52 bp deletion and 5 bp insertion affect the oxidative stress response and whether aberrant response could indicate a pathogenic mechanism in mutant *CALR*-mediated cellular transformation. Subsequently, we tested the effect of the *CALR* mutation on the capacity of cells to respond to oxidative stress-induced DNA damage. Data on the role of mutant calreticulin in oxidative stress-induced apoptosis are limited; therefore, we intended to analyze whether the impaired ability to reduce oxidative stress entails a different ability to induce apoptosis. The cell cycle involvement in malignant processes is determined; unfortunately, to the extent of our knowledge, no data shows the impact of mutated calreticulin on cell cycle progression in the context of oxidative stress. Thus, we also aimed to evaluate cell cycle distribution after oxidative stress induction in *CALR*-mutated cells.

## 2. Results

### 2.1. The Dependence of CALR Del52, CALR Ins5 Cells on JAK/STAT, PI3K/Akt/mTOR, and Hedgehog Signaling

**Figure 1:**
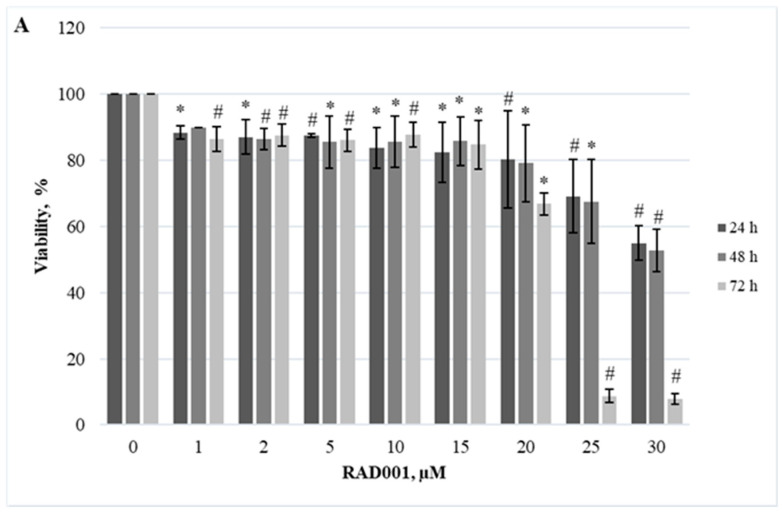

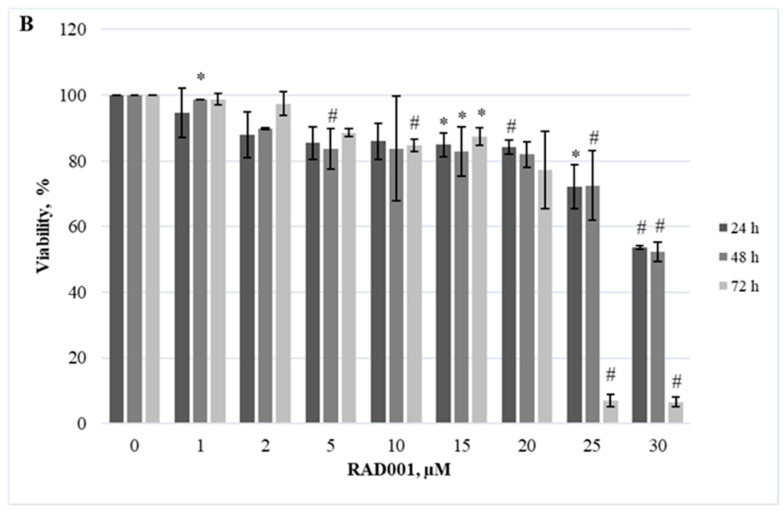
*CALR* Del52 cell line viability reduction by RAD001 treatment. *CALR* Del52 cells were treated with varying concentrations of RAD001 for 24, 48, and 72 h before alamarBlue (**A**) and trypan exclusion assays (**B**) were performed. * *p* < 0.05 vs. DMSO-treated control. # *p* < 0.01 vs. DMSO-treated control.

**Figure 2:**
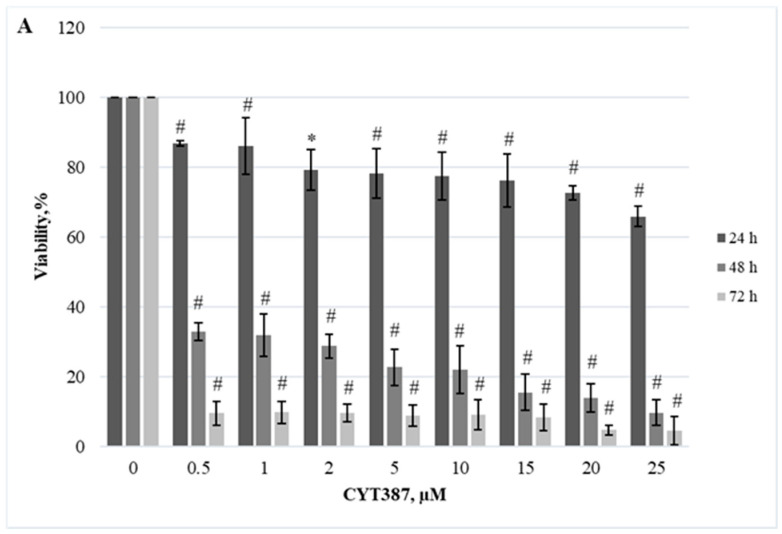

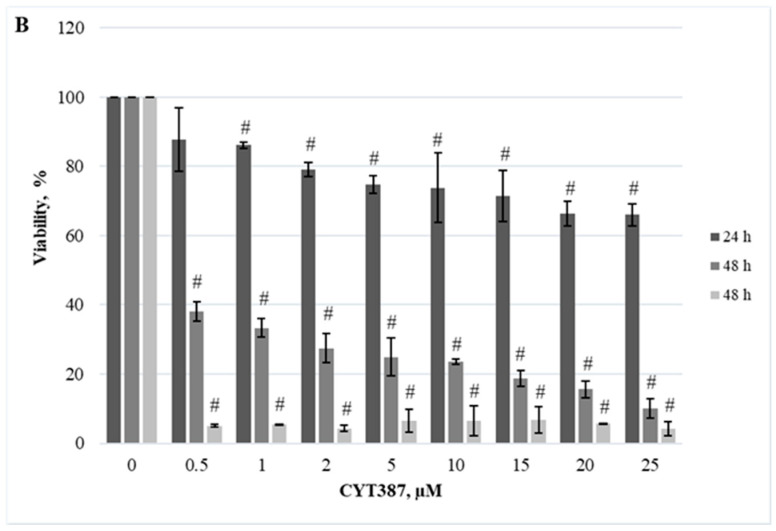
Reduction of *CALR* Del52 cell line viability by CYT387 treatment. *CALR* Del52 cells were treated with varying concentrations of CYT387 for 24, 48, and 72 h before alamarBlue (**A**) and trypan exclusion assays (**B**) were performed. * *p* < 0.05 vs. DMSO-treated control. # *p* < 0.01 vs. DMSO-treated control.

**Figure 3:**
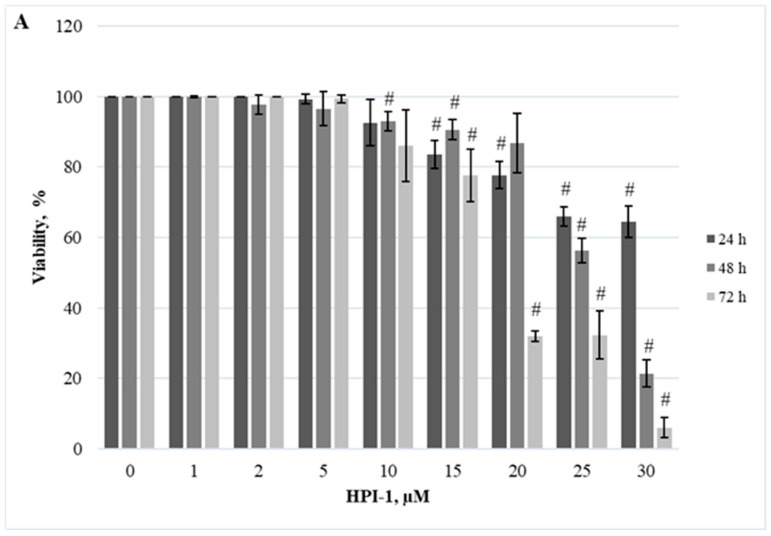

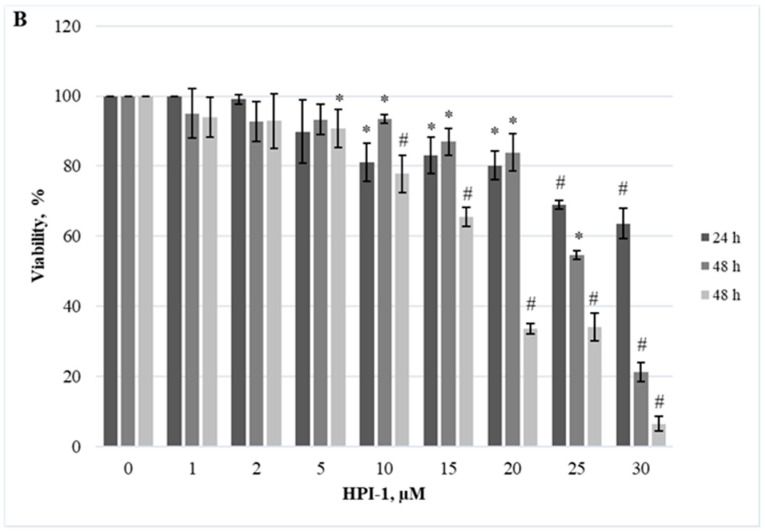
Reduction of *CALR* Del52 cell line viability by HPI-1 treatment. *CALR* Del52 cells were treated with varying concentrations of HPI-1 for 24, 48, and 72 h before alamarBlue (**A**) and trypan exclusion assays (**B**) were performed. * *p* < 0.05 vs. DMSO-treated control. # *p* < 0.01 vs. DMSO-treated control.

**Figure 4:**
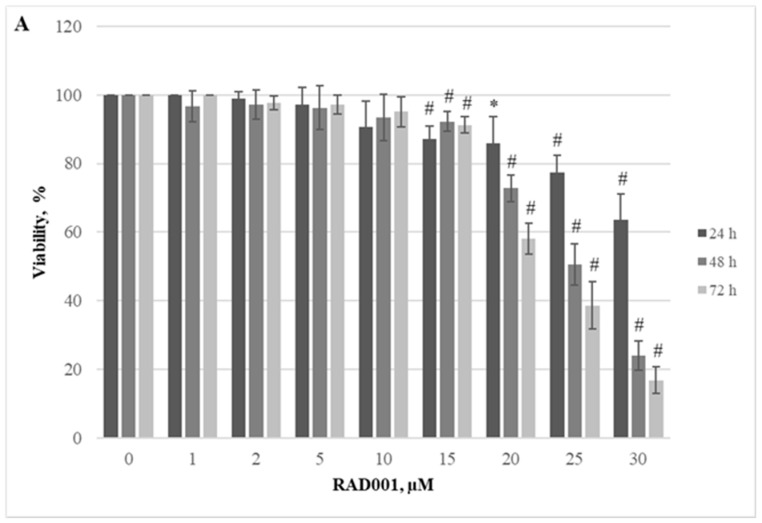

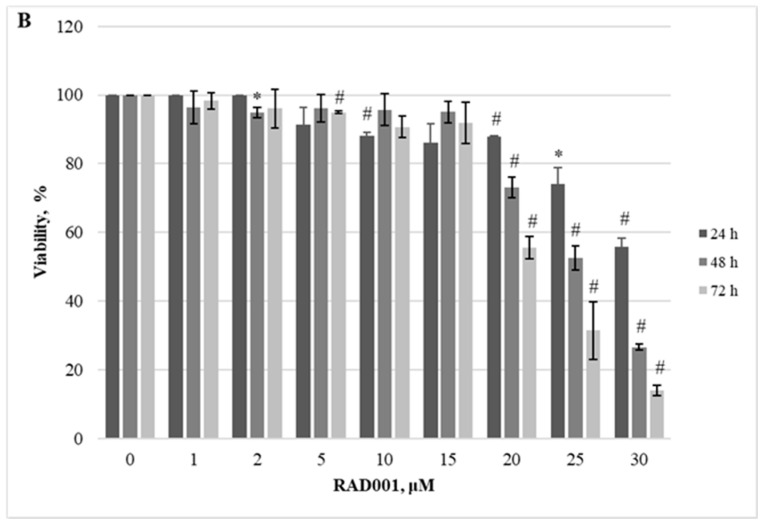
*CALR* Ins5 cell line viability reduction by RAD001 treatment. *CALR* Ins5 cells were treated with varying concentrations of RAD001 for 24, 48, and 72 h before alamarBlue (**A**) and trypan exclusion assays (**B**) were performed. * *p* < 0.05 vs. DMSO-treated control. # *p* < 0.01 vs. DMSO-treated control.

**Figure 5:**
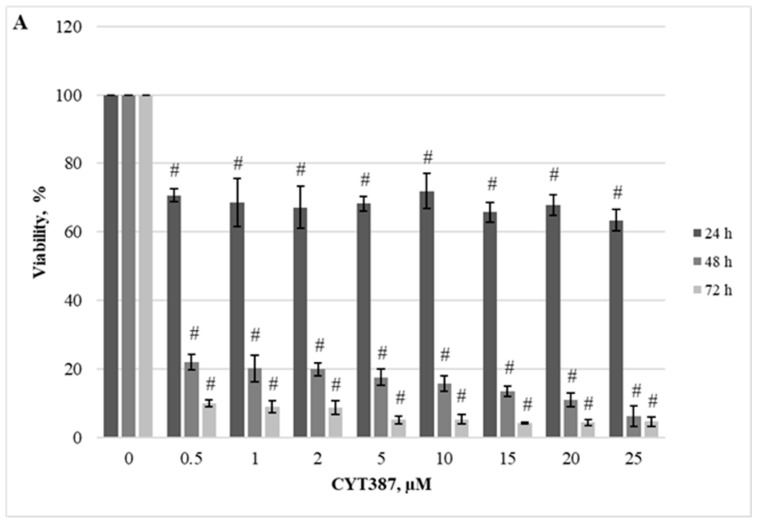

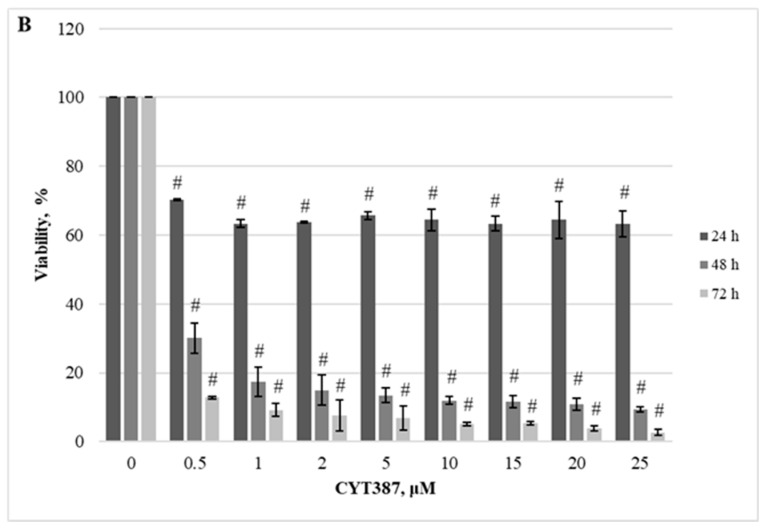
Reduction of *CALR* Ins5 cell line viability by CYT387 treatment. *CALR* Ins5 cells were treated with varying concentrations of CYT387 for 24, 48, and 72 h before alamarBlue (**A**) and trypan exclusion assays (**B**) were performed. # *p* < 0.01 vs. DMSO-treated control.

**Figure 6:**
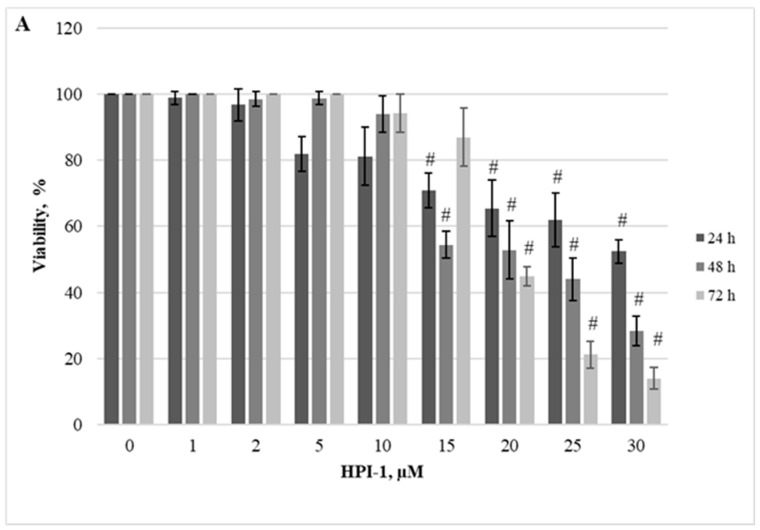

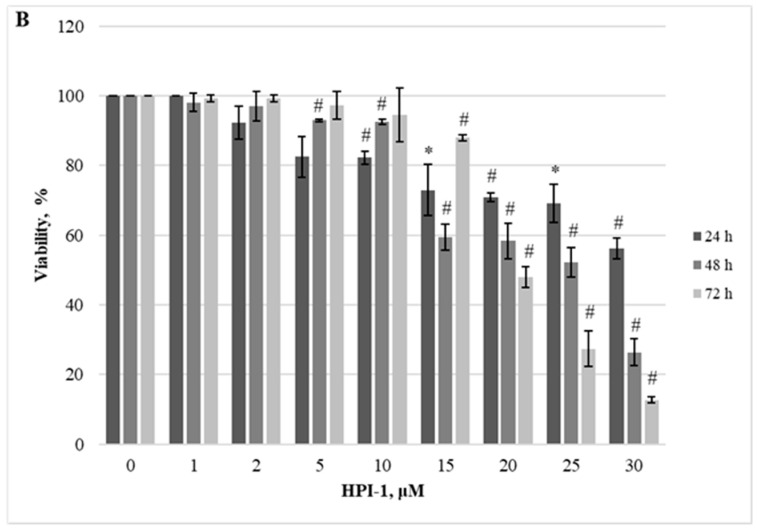
Reduction of *CALR* Ins5 cell line viability by HPI-1 treatment. *CALR* Ins5 cells were treated with varying concentrations of HPI-1 for 24, 48, and 72 h before alamarBlue (**A**) and trypan exclusion assays (**B**) were performed. * *p* < 0.05 vs. DMSO-treated control. # *p* < 0.01 vs. DMSO-treated control.

Herein, we demonstrated the dependence of different cellular models on the JAK/STAT, PI3K/Akt/mTOR, and Hedgehog signaling pathways. Firstly, we evaluated the effect of RAD001 (against mTOR), CYT387 (against JAK1/2), and HPI-1 (against Hedgehog signaling) inhibitors as a single agent. A reduction in cell viability was observed in all tested cell lines using alamarBlue and trypan exclusion assays. Our study results indicate that inhibitors progressively reduced cell viability as the concentration and exposure duration increased. The dose−response study demonstrated that agents targeting JAK/STAT, PI3K/Akt/mTOR, and Hedgehog signaling pathways exert significant inhibition of *CALR*-mutated cells (Figure 1, Figure 2, Figure 3, Figure 4, Figure 5 and Figure 6). It is important to mention that the results related to SET-2 and UT-7 cells’ antiproliferative response to specific inhibitors were published previously in our article [84].

No statistically significant differences were found between the IC50 values of the inhibitors when *CALR* Del52 cells were treated for 24 h (*p* > 0.05). However, *CALR*-mutated cells were extremely sensitive, demonstrating low IC50 values for JAK/STAT inhibitor CYT387 compared to RAD001 after 48 h and 72 h exposure (0.84 vs. 41.05, *p* = 0.0001 and 0.34 vs. 19.32, *p* = 0.0001, respectively) when cell viability was assessed with trypan blue exclusion assay. This difference was confirmed by the alamarBlue test (2.71 vs. 42.32, *p* = 0.001, and 0.39 vs. 18.58, *p* = 0.0001). *CALR*-mutated cells were less sensitive to HPI-1 inhibitor compared to CYT387 after 48 and 72 h treatment (26.93 vs. 0.84, *p* = 0.0001 and 17.44 vs. 0.34, *p* = 0.0001) (according to trypan blue assay results). The results were confirmed by the alamarBlue test, where IC50 values were 27.66 vs. 2.71 (*p* = 0.012) and 18.36 vs. 0.39 (*p* = 0.0001). Moreover, a statistically significant difference was found between the RAD001 and HPI-1 treatments. The trypan blue exclusion assay data showed that cells harboring the *CALR* 52 bp deletion were more sensitive to HPI-1 compared to RAD001 (26.93 vs. 41.05, *p* = 0.02 (48 h) and 17.44 vs. 19.32, *p* = 0.002 (72 h)). However, this difference was not confirmed by the alamaBlue test (*p* > 0.05) (Table S1, Supplementary Material). No statistically significant differences were found between CYT387, RAD001, and HPI-1 inhibitors’ IC50 values when *CALR* Ins5 cells were treated for 24 h (*p* > 0.05). However, dose-response studies with trypan blue exclusion assay showed that *CALR* Ins5 cells were more sensitive to CYT387 after 48 and 72 h exposure, compared to RAD001 treatment (0.47 vs. 27.01, *p* = 0.0001 and 0.32 vs. 21.01, *p* = 0.0001, respectively). The difference was also confirmed by the alamarBlue test (0.80 vs. 25.92, *p* = 0.0001, and 0.61 vs. 22.31, *p* = 0.0001). Furthermore, a statistically significant difference was found between the CYT387 and HPI-1 treatments. The Hedgehog signaling inhibitor HPI-1 had less inhibitory potential than CYT387, as a trypan blue exclusion assay data showed. After 48 and 72 h of treatment, IC50 values were as follows: 22.94 vs. 0.47, *p* = 0.0001, and 20.09 vs. 0.32, *p* = 0.0001, respectively. The alamarBlue test demonstrated similar results: 21.72 vs. 0.80, *p* = 0.0001, and 19.61 vs. 0.61, *p* = 0.0001. A statistically significant difference was found between RAD001 and HPI-1 treatments, but only in the data obtained from the alamarBlue assay. The data showed that cells harboring the *CALR* 5 bp insertion were more sensitive to HPI-1 after 48 and 72 h exposure, compared to RAD001 (21.72 vs. 25.92, *p* = 0.004 and 19.61 vs. 22.31, *p* = 0.010) (Table S1, Supplementary Material). Assessing agent sensitivity among cell lines, data from this and a previously published study [84] show that cells with *CALR* mutations are more sensitive to the tested inhibitors than the control UT-7 cell line.

Briefly, our previous study provided a broad overview of MPN-related signaling pathways and conducted a dose-response analysis of RAD001, CYT387, and HPI-1 in SET-2 and UT-7 cells. We found that SET-2 cells expressing the *JAK2* p. V617F mutation showed increased sensitivity toward the tested agents compared to the control UT-7 cells. Our data indicate that the cell line expressing *JAK2* p.V617F is exquisitely sensitive to CYT387 as a single agent, showing proliferation arrest. We found that *JAK2* mutated cells were more sensitive to inhibition of the PI3K/Akt/mTOR and Hh pathways than the wild-type counterpart UT-7 cell line. CYT387 presented lower IC50 values compared to RAD001 and HPI-1 in the UT-7 cell line after 24, 48, and 72 h of treatment [84].

To sum up, our data indicate that the cells expressing *CALR* mutations were exquisitely sensitive to CYT387 as a single agent, showing proliferation arrest. Moreover, we found that the SET-2 cell line, *CALR* Del52, and *CALR* Ins5 cells were more sensitive to the inhibition of PI3K/Akt/mTOR and Hedgehog signaling pathways than the wild-type counterpart UT-7 cell line.

### 2.2. The Expression of JAK/STAT, PI3K/Akt/mTOR, and Hedgehog Signaling Pathways-Related Genes in CALR Del52, CALR Ins5, SET-2 and UT-7 Cells

In the next step of our study, we aimed to analyze the expression of JAK/STAT, PI3K/Akt, and Hedgehog signaling-related genes, which are the main components of these signaling pathways. *STAT1* and *STAT5* are related to JAK/STAT signaling, *EIF4EBP1* and *RPS6KB1* are the main components of the PI3K/Akt/mTOR pathway, and *PTCH1* expression is associated with Hedgehog signaling. To assess the changes in gene expression, the RT-qPCR method was applied.

**Figure 7:**
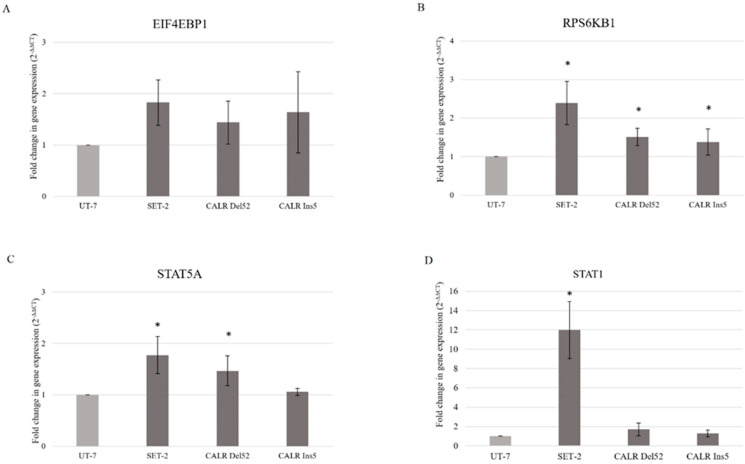
Gene expression in *CALR* Del52, *CALR* Ins5, and SET-2 cells. (**A**) *EIF4EBP1*, (**B**) *RPS6KB1*, (**C**) *STAT5A*, and (**D**) *STAT1* relative expression levels (2^−ΔΔCt^) were normalized to *ACTB* gene expression as an endogenous control. Bar graphs represent the mean of three independent experiments. Error bars show the standard deviation from the mean. The differences between the UT-7 cell line, which served as a *JAK2* and *CALR* wild-type control, and SET-2 and *CALR*-mutated cells were evaluated using an independent sample *t*-test. Gene expression, which is significantly different from the control at *p* < 0.05, is represented as *.

It was found that there were significant differences in target gene expression between *CALR* Del52, *CALR* Ins5, SET-2, and UT-7 cells, which served as control cells with wild-type *JAK2* and *CALR*. *RPS6KB1* gene was found to be increased in the *JAK2* mutated SET-2 cell line by 2.390-fold (*p* = 0.046) compared to that in the UT-7 cell line (Figure 7B). *RPS6KB1* gene was also upregulated in *CALR* Del52 and *CALR* Ins5 cells (1.510-fold, *p* = 0.048 and 1.382-fold, *p* = 0.022, respectively) (Figure 7B). Moreover, *STAT5A* gene expression was found to be increased by 1.775-fold (*p* = 0.050) in SET-2 and by 1.466-fold (*p* = 0.037) in *CALR* Del52 cells compared to the UT-7 cell line (Figure 7C). The expression of the *STAT1* gene was significantly increased by 11.985-fold (*p* = 0.026) in the SET-2 cell line compared to that in wild-type UT-7 cells (Figure 7D). The *EIF4EBP1* gene showed a tendency for upregulation; however, the change in gene expression did not reach the significance level (Figure 7A). No *PTCH1* expression was detected in any of the tested cell lines.

It can be concluded that the expression of JAK/STAT and PI3K/Akt/mTOR signaling pathways-related genes is increased in *CALR* Del52, *CALR* Ins5, and SET-2 cells.

### 2.3. Phosphorylation Level of JAK/STAT and PI3K/Akt/mTOR Signaling Pathways-Related Proteins in CALR Del52, CALR Ins5, SET-2, and UT-7 Cells

Here, we aimed to determine whether the increase in mRNA corresponded with elevated total and phosphorylated protein levels in the tested cell lines. Western blot and densitometry analyses were performed to evaluate the change in protein level.

**Figure 8:**
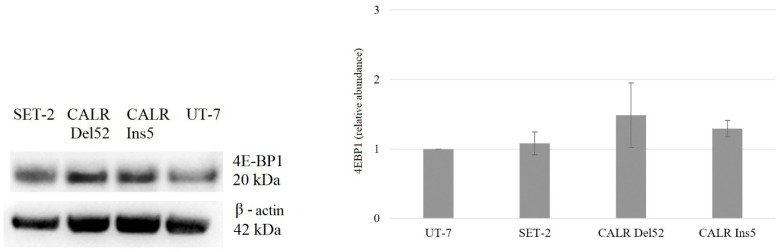
Changes in 4E-BP1 protein levels in *CALR* Del52, *CALR* Ins5, and SET-2 cell lines. Representative pictures of protein bands on the left of the figure are obtained from Western blot membranes and show protein expression levels. Densitometric quantification of total 4E-BP1 levels was normalized to that of β-actin for fold-change calculations. Bar graphs represent the mean of at least three independent experiments. Error bars show the standard deviation from the mean. The differences in protein expression between the UT-7 cell line, which served as a *JAK2* and *CALR* wild-type control, and SET-2 and *CALR*-mutated cells, were evaluated using an independent sample *t*-test.

We focused on molecules that are the main targets of mTORC1, i.e., *EIF4EBP1* and *RPS6KB1*, and mRNA analysis showed significantly increased expression of *RPS6KB1*. However, Western blot and densitometry analysis revealed that *CALR* Del52, *CALR* Ins5, SET-2, and UT-7 cells lack total and phosphorylated S6K1 protein (encoded by the *RPS6KB1* gene) expression levels (data not presented). Therefore, for further analysis, only phosphorylated 4E-BP1 was used as a marker of mTORC1 activity. It was determined that SET-2 cells harboring *JAK2* mutation, *CALR* Del52, and *CALR* Ins5 cells had upregulated levels of total 4E-BP1 protein (Figure 8).

**Figure 9:**
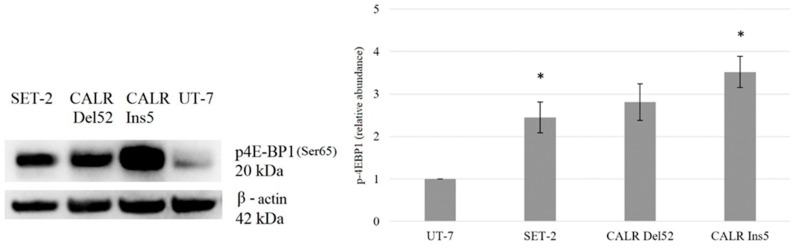
Induction of the phosphorylation of 4E-BP1 in *CALR* Del52, *CALR* Ins5, and SET-2 cell lines. Representative pictures of protein bands on the left of the figure are obtained from Western blot membranes and show induced activation of 4E-BP1. Densitometric quantification of phosphorylated 4E-BP1 levels was normalized to total 4E-BP1 for fold-change calculations. The bar graphs represent the mean of at least three independent experiments. Error bars show the standard deviation from the mean. The differences in protein levels between the UT-7 cell line, which served as a *JAK2* and *CALR* wild-type control, and SET-2 and *CALR*-mutated cells, were evaluated using an independent sample *t*-test. Protein levels significantly different from the control (*p* < 0.05) are represented as *.

Western blot data revealed a significant 2.445-fold (*p* = 0.005) and 3.581-fold (*p* = 0.001) increase in phosphorylation of 4E-BP1 in SET-2 and *CALR* Ins5 cells (compared to control UT-7 cell line), respectively (Figure 9).

**Figure 10:**
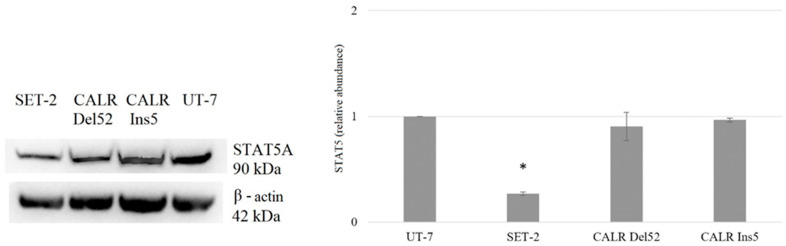
Changes in STAT5A protein levels in *CALR* Del52, *CALR* Ins5, and SET-2 cell lines. Representative pictures of protein bands on the left of the figure are obtained from Western blot membranes and show protein expression. Densitometric quantification of total STAT5A levels was normalized to that of β-actin for fold-change calculations. The bar graphs represent the mean of at least three independent experiments. Error bars show the standard deviation from the mean. The differences in protein levels between the UT-7 cell line, which served as a *JAK2* and *CALR* wild-type control, and SET-2 and *CALR*-mutated cells, were evaluated using an independent sample *t*-test. Protein level significantly different from the control at *p* < 0.05 is represented as *.

Further, we aimed to analyze whether the JAK/STAT signaling pathway targets, STAT5 and STAT1, are affected in cells harboring *CALR* and *JAK2* mutations. The total STAT5A protein level (Figure 10) was significantly reduced in SET-2 cells (3.704-fold, *p* = 0.010), whereas STAT5A expression in *CALR*-mutated cells did not differ from that in the UT-7 control cell line.

**Figure 11:**
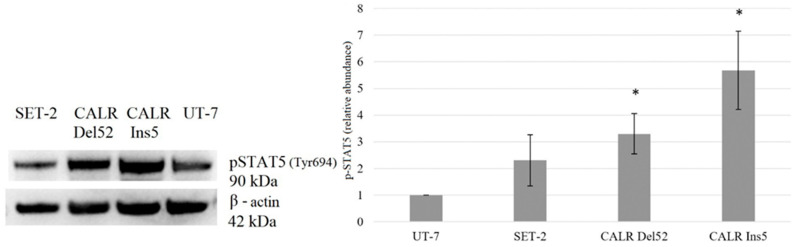
Induction of the phosphorylation of STAT5A in *CALR* Del52, *CALR* Ins5, and SET-2 cell lines. Representative pictures of protein bands on the left of the figure are obtained from Western blot membranes and show induced activation of STAT5A. Densitometric quantification of phosphorylated STAT5A levels was normalized to total STAT5A for fold-change calculations. The bar graphs represent the mean of at least three independent experiments. Error bars show the standard deviation from the mean. The differences in protein levels between the UT-7 cell line, which served as a *JAK2* and *CALR* wild-type control, and SET-2 as well, as *CALR*-mutated cells, were evaluated using an independent sample *t*-test. Protein level significantly different from the control at *p* < 0.05 is represented as *.

Next, we evaluated the phosphorylated STAT5A protein levels. Our data revealed that the phosphorylation of STAT5A was increased in cells carrying mutated *CALR*. Western blot analysis indicated a 3.30-fold (*p* = 0.050) increase in STAT5A phosphorylation in *CALR* Del52 cells. Moreover, *CALR* Ins5 cells showed a 5.677-fold (*p* = 0.046) increase in STAT5 phosphorylation (Figure 11).

**Figure 12:**
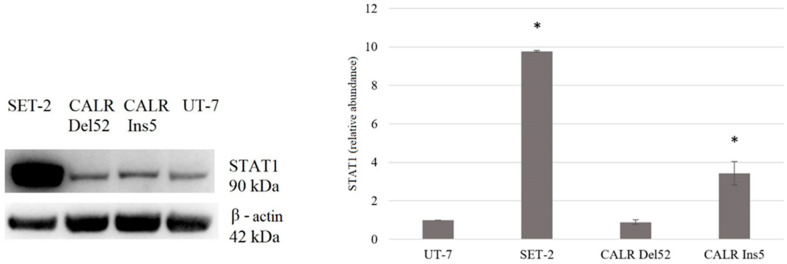
Changes in STAT1 protein levels in *CALR* Del52, *CALR* Ins5, and SET-2 cell lines. Representative pictures of protein bands on the left of the figure are obtained from Western blot membranes and show protein expression. Densitometric quantification of total STAT1 levels was normalized to β-actin levels for fold-change calculations. The bar graphs represent the mean of at least three independent experiments. Error bars show the standard deviation from the mean. Differences in protein levels between the UT-7 control cell line and SET-2, as well as *CALR*-mutated cells, were evaluated using an independent sample *t*-test. Protein level significantly different from the control at *p* < 0.05 is represented as *.

The upregulated total STAT1 levels were found only in SET-2 and *CALR* Ins5 cells (9.773-fold, *p* = 0.002 and 3.429-fold, *p* = 0.030, respectively) (Figure 12).

**Figure 13:**
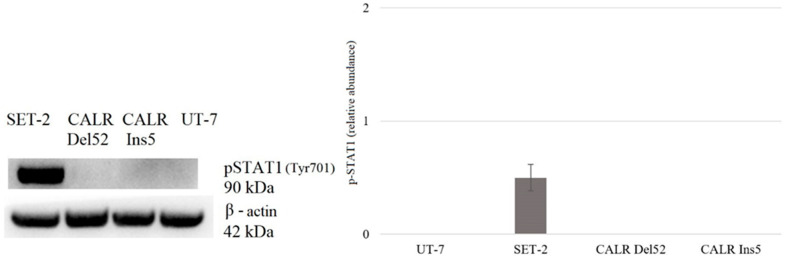
Changes in STAT1 protein phosphorylation in *CALR* Del52, *CALR* Ins5, and SET-2 cell lines. Representative pictures of protein bands on the left of the figure are obtained from Western blot membranes and show induced activation of STAT1. Densitometric quantification of phosphorylated STAT1 levels was normalized to total STAT1 for fold-change calculations. The bar graphs represent the mean of at least three independent experiments. The protein level differences between the UT-7 cell line, which served as a *JAK2* and *CALR* wild-type control, and SET-2 as well, as *CALR*-mutated cells, were evaluated using an independent sample *t*-test. Error bars show the standard deviation from the mean.

Lastly, the expression level of phosphorylated STAT1 was evaluated. Western blot revealed a detectable reduction in STAT1 phosphorylation in SET-2 cells. However, no detectable phosphorylation of STAT1 protein was found in *CALR* Del52 and *CARL* Ins5 cells (Figure 13).

### 2.4. Effect of CALR Mutations on the Physiological Calreticulin Functions

As mentioned before, calreticulin is a major chaperone in the ER and plays a role in several cellular processes, e.g., control of protein folding, calcium homeostasis, and cellular stress response. However, there is little data that describes the effects of *CALR* mutations on the physiological function of calreticulin. It has been demonstrated that cells overexpressing *CALR* show increased sensitivity to ER and oxidative stress [40,83]. Thus, we aimed to determine how *CALR* 52 bp deletion and 5 bp insertion affect the oxidative stress response and whether an abnormal response could indicate a pathogenic mechanism in mutant *CALR*-mediated cellular transformation. To analyze the effect of common *CALR* mutations on the response to oxidative stress, all tested cells were exposed to hydrogen peroxide (H_2_O_2_) for 24 h. Subsequently, the cells were incubated for an additional 24 h to reduce the ROS accumulation induced by H_2_O_2_. The quantitative measurement of cells undergoing oxidative stress was performed using the cell analyzer Muse with the corresponding kit, according to the manufacturer’s protocol.

**Figure 14:**
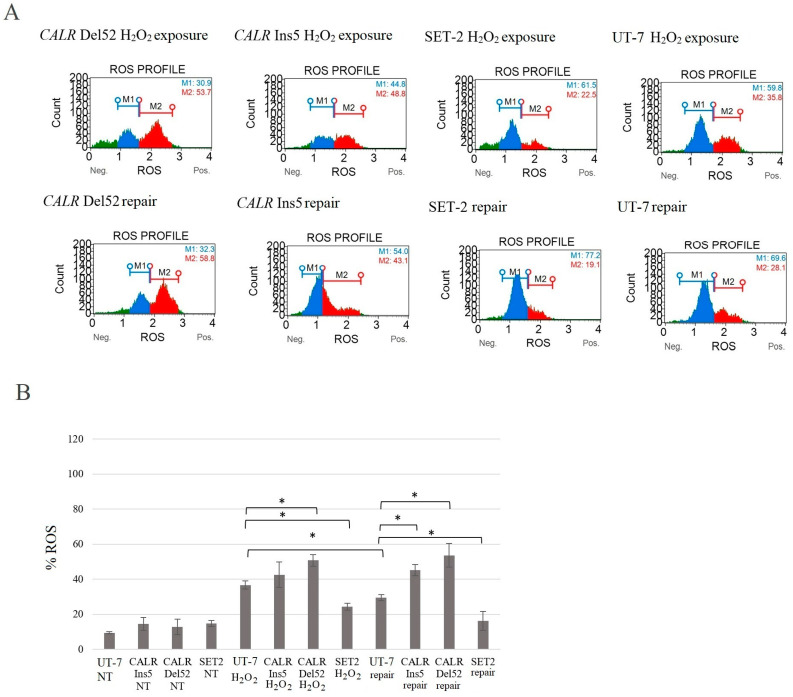
Results of ROS level analysis in *CALR* Del52, *CALR* Ins5, SET-2, and UT-7 cells after 24 h of exposure to H_2_O_2_ and 24 h of repair. (**A**) Representative ROS profile histograms are shown. (**B**) The graph represents the percentage of positive ROS in different cell lines. Values represent the mean and a standard deviation of at least three experiments performed in triplicate. An asterisk represents *p* < 0.05 vs. control.

It was revealed that after H_2_O_2_ treatment, *CALR* Del52 cells showed elevated levels of ROS compared to the UT-7 cell line harboring wild-type *CALR* (50.8 ± 3.3% vs. 36.7 ± 2.3%, *p* = 0.005). Contrary, *JAK2* p.V617F positive SET-2 cell line showed relatively low ROS levels compared to the control UT-7 cells (24.4 ± 2.0% vs. 36.7 ± 2.3%, *p* = 0.0001) (Figure 14). We showed that these differences are more remarkable after 24 h of repair. *CALR* Del52 and *CALR* Ins5 cells could not reduce intracellular ROS levels, while the control UT-7 cells were able to counteract ROS accumulation (53.7 ± 6.8% vs. 29.5 ± 1.6%, *p* = 0.009 and 45.1 ± 3.2% vs. 29.5 ± 1.6%, *p* = 0.006, respectively). The SET-2 cell line efficiently decreased the percentage of ROS-positive cells compared to the control (16.2 ± 5.3% vs. 29.5 ± 1.6%, *p* = 0.038) (Figure 14).

Next, we aimed to study whether *CALR* 52 bp deletion and 5 bp insertion can affect the efficiency of repairing the DNA damage induced by oxidative stress. The cells expressing the wild-type *CALR* and *JAK2*, as well as mutated cells, were exposed to H_2_O_2_ for 24 h. Subsequently, the cells were given an additional 24 h to repair the DNA damage induced by H_2_O_2_ treatment. pATM and pH2AX were measured using cytometry and the Muse Multi-Color DNA Damage Kit. The phosphorylation of ATM and H2AX was taken together to analyze the results.

**Figure 15:**
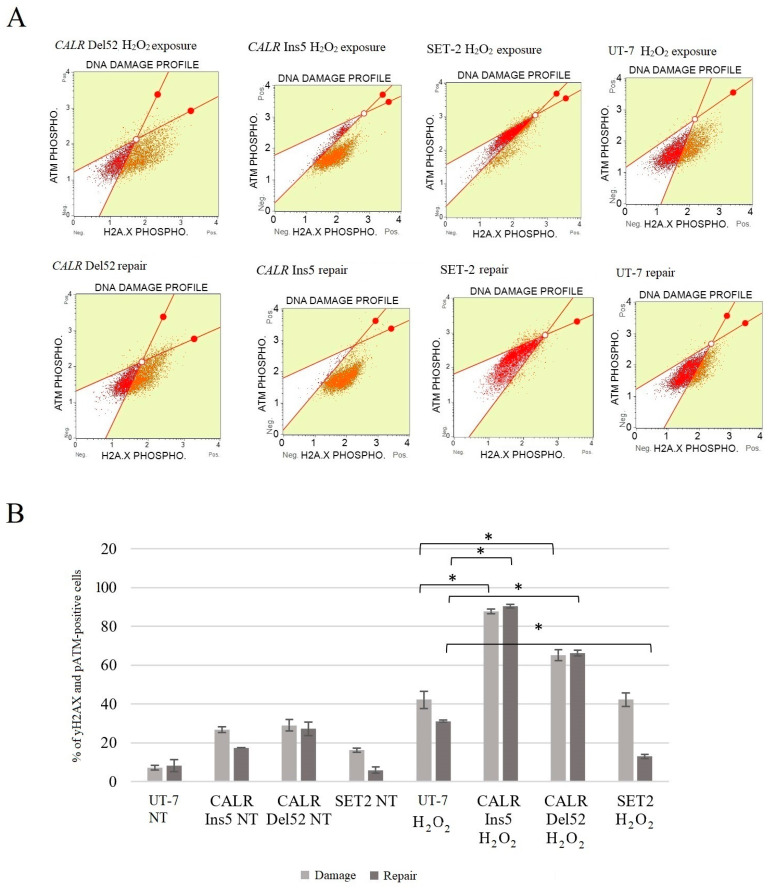
*CALR* mutations impair oxidative stress-induced DNA damage repair. DNA damage in SET-2, *CALR* Del52, *CALR* Ins5, and UT-7 was detected after 24 h of exposure to H_2_O_2_ and 24 h of repair using the Muse Multi-Color DNA Damage Kit. (**A**) Representative scatterplots of pATM and pH2AX profiles in *JAK2*-mutated, *CALR*-mutated, and wild-type cells. (**B**) The graph shows the percentage of cells expressing phosphorylated ATM and H2AX. Values show the mean with a standard deviation of at least three experiments performed in triplicate. An asterisk represents *p* < 0.05 vs. control.

Our study demonstrated that *CALR* Del52 cells are characterized by statistically significant higher levels of DNA damage compared to the control UT-7 cell line (65.2 ± 2.8% vs. 42.3 ± 3.5%, *p* = 0.037). Moreover, *CALR* Ins5 cells showed a higher level of phosphorylated ATM and H2AX compared to *CALR* wild-type cells (87.6 ± 1.2% vs. 42.3 ± 3.5%, *p* = 0.032). These differences were exceptional after cells were given an additional 24 h to repair oxidative stress-induced DNA damage. After 24 h of repair, the control UT-7 cell line was able to repair DNA damage induced by H_2_O_2_ treatment. On the contrary, *CALR* Del52 and *CALR* Ins5 cells were not able to repair DNA damage, as evidenced by the pATM and pH2AX levels (66.3 ± 1.5% vs. 31.2 ± 0.5%, *p* = 0.011 and 90.5 ± 0.9% vs. 31.2 ± 0.5%, *p* = 0.001, respectively). The SET-2 cell line with *JAK2* mutation showed moderate DNA damage after H_2_O_2_ treatment and was able to repair it (13.1 ± 0.9% vs. 31.2 ± 0.5%, *p* = 0.006). According to our study results, cells carrying *CALR* 52 bp deletion and 5 bp insertion had a lower capability to repair DNA damage induced by oxidative stress (Figure 15).

Calreticulin plays a role in a variety of biological processes, including cell death. Nevertheless, data on the effects of mutant calreticulin on oxidative stress-induced apoptosis are limited. Therefore, we intended to analyze whether the impaired ability to reduce oxidative stress entails a different ability to induce cell death. Apoptosis was assessed using the Muse Annexin V and Dead Cell Kit.

**Figure 16:**
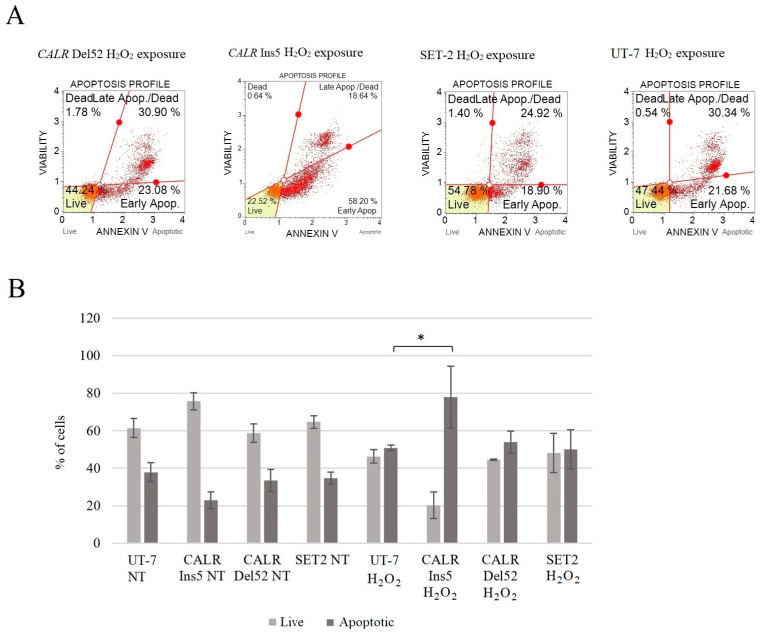
An increase in apoptosis level in *CALR* Ins5 cells after H_2_O_2_-induced oxidative stress. The evaluation of apoptosis levels in the tested cells was performed after 24 h of exposure to H_2_O_2_ using the Muse Annexin V and Dead Cell Kit. (**A**) Representative scatter plots of the apoptosis profile are shown. (**B**) The graph shows the percentage of apoptotic cells. Values show the mean with a standard deviation of at least three experiments performed in triplicate. An asterisk represents *p* < 0.05 vs. control.

It was determined that apoptosis was significantly increased in cells harboring the *CALR* 5 bp insertion (78.0 ± 16.4% vs. 50.9 ± 1.4%, *p* = 0.012). In addition, the apoptosis level was higher (53.8 ± 5.9%, *p* = 0.063) in *CALR* Del52 cells, even though it was not statistically significant. On the contrary, SET-2 cells showed a lower level of apoptosis compared to the control UT-7 cell line; however, the difference was not statistically significant (50.0 ± 10.4%, *p* = 0.887 vs. 50.9 ± 1.4%, *p* = 0.667) (Figure 16).

Lastly, we aimed to analyze the cell cycle distribution after oxidative stress induction in *CALR*-mutated and *JAK2*-mutated cells. Cell cycle machinery is seen in essentially all tumor types and represents a driving force of tumorigenesis; however, to the best of our knowledge, no data show the impact of mutated *CALR* on cell cycle progression in the context of oxidative stress. The analysis of the cell cycle distribution was carried out after 24 h of exposure to H_2_O_2_ using the Muse Cell Cycle Kit.

**Figure 17:**
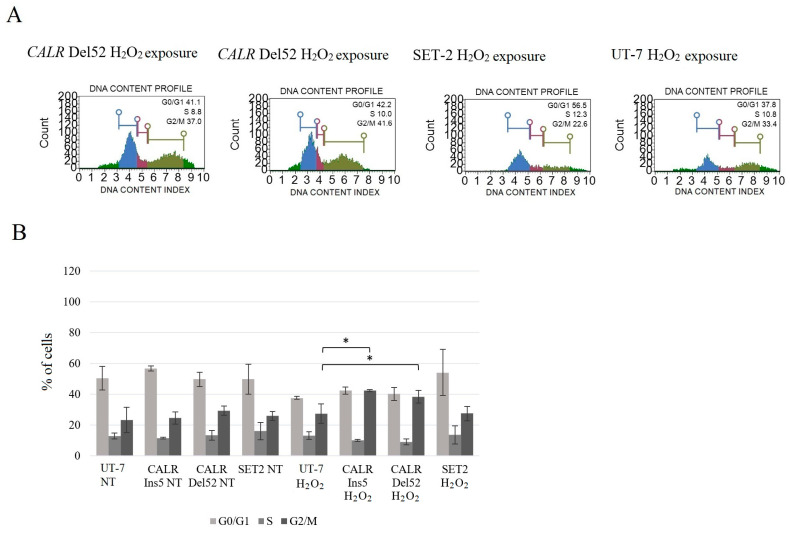
*CALR*-mutated cells cycle arrest at the G2/M phase after H_2_O_2_-induced oxidative stress. The analysis of the cell cycle was performed after 24 h of exposure to H_2_O_2_ using a Muse Cell Cycle Kit. (**A**) Representative histograms of cell cycle distribution in the tested cell lines. (**B**) The graph represents the percentage of cell cycle distribution. Values show the mean with a standard deviation of at least three experiments performed in triplicate. An asterisk represents *p* < 0.05 vs. control.

It was determined that cells harboring *CALR* 52 bp deletion showed cell cycle arrest at the G2/M phase (Figure 17) compared to the control UT-7 cell line after H_2_O_2_ treatment (38.3 ± 4.2% vs. 27.3 ± 6.3%, *p* = 0.038). Furthermore, the H_2_O_2_ exposure was shown to arrest the cell cycle at the G2/M phase in *CALR* Ins5 cells (42.4 ± 0.7% vs. 27.3 ± 6.3%, *p* = 0.015). Cell cycle changes were not found in the SET-2 cell line carrying the *JAK2* mutation. Our data suggest that the *CALR* mutation affects not only apoptosis levels but also cell cycle arrest at the G2/M phase after H_2_O_2_-induced oxidative stress. It is important to note that cell cycle arrest at G2/M blocks cell cycle progression, providing the potential for DNA damage repair [85].

## 3. Discussion

The role of mutated calreticulin in various biological processes and its association with MPN have recently been analyzed in several studies. However, the involvement of the *CALR* 52 bp deletion and *CALR* 5 bp insertion in the pathogenesis of MPNs has not been fully investigated, and the final conclusions have not been presented. Thus, in our study, we intended to contribute to a deeper analysis of the molecular mechanisms of mutated *CALR*. In the first part of our research, we aimed to determine the mutant *CALR* association with JAK/STAT and alternative PI3K/Akt/mTOR and Hedgehog signaling pathways that are related to MPNs.

The dependence of *CALR* Del52, *CALR* Ins5, SET-2, and UT-7 cells on the aforementioned signaling pathways was assessed using the specific inhibitors RAD001 (against mTOR), CYT387 (against JAK1/2), and HPI-1 (against Gli1/2). Subsequently, target gene expression and protein levels of JAK/STAT, PI3K/Akt/mTOR, and Hedgehog signaling pathway components were determined. In more detail, the results that address the antiproliferative effect of inhibitors on SET-2 and UT-7 cell viability have already been published in our previous article [84], but these results are inseparable from our current study research; therefore, a brief discussion and comparison of the previously obtained results is necessary. We found that all specific inhibitors progressively reduced the number of viable cells as the concentration and exposure time increased. It was observed that cells carrying the *CALR* mutation were less resistant to the CYT387 inhibitor than SET-2 cells expressing *JAK2* p.V617F and UT-7 cells with wild-type *JAK2* and *CALR*. Our results indicate that CYT387 inhibited the proliferation of cells harboring *CALR* mutations with the lowest IC50 values compared to RAD001 and HPI-1. Furthermore, *STAT5A* gene expression was elevated in cells with *CALR* 52 bp deletion. Consistent with these data, Western blot analysis showed that phosphorylated STAT5 protein levels were also increased in *CALR* Del52 cells. Although significant *STAT5* and *STAT1* gene expression was not detected, protein quantification revealed that STAT1 and phosphorylated STAT5 levels increased in *CALR* Ins5 cells. Our results agree with those of previous studies showing that the JAK/STAT signaling pathway is activated in cells harboring *CALR* mutations. However, only a few studies have performed dose-response experiments using *CALR*-mutated cells and JAK/STAT inhibitors [10,35,52]. The functional effects of mutated *CALR* were analyzed in murine Ba/F3 and 32D cells [10,52], which were transduced with *CALR* mutations and showed significant accumulation in the absence of interleukin-3. Moreover, transduced cells also demonstrated sensitivity to the JAK2 kinase inhibitor, suggesting that the interleukin-3-independent growth of *CALR*-mutated cells depends on the JAK/STAT pathway. Increased activation of JAK/STAT signaling pathways was also found after analysis of the phosphorylation of STAT5. However, other researchers have reported distinct JAK/STAT signaling signatures in *CALR*-mutated cells. It was determined that MARIMO cells carrying the *CALR* 61 bp deletion were resistant to JAK1/2 inhibitor, compared to *JAK2* p.V617F-mutant cells. The MARIMO cell line independence on JAK/STAT signaling was confirmed with Western blot analysis, where cells showed reduced levels of JAK2, phosphorylated JAK2, STAT5, phosphorylated STAT5, STAT1, phosphorylated STAT1, STAT3, and phosphorylated STAT3. Nevertheless, *JAK2* transcript levels in MARIMO cells were similar to those in *JAK2*-mutated cells, suggesting decreased translation or increased degradation of JAK2 protein [35]. Recently, it has been suggested that the consequences of *CALR* 52 bp deletion and 5 bp insertion for megakaryopoiesis are likely to be mediated by the MAPK and STAT5 pathways accompanied by MPL-dependent activation. The mentioned results were obtained in a complex analysis with CD34^+^ cells infected with lentiviral constructs expressing mutant *CALR*, MARIMO, and murine cells, Ba/F3 and 32D harboring *CARL* 52 bp deletion and 5 bp insertion [34]. Such differences in the described results could be due to the different cell types used for the experiments, their mutational status, cultivation conditions, and assays that were carried out. For example, the origin of Ba/F3 and 32D cells is different from that of cells that carry *CALR* mutations in humans, i.e., the mentioned suspension cells originated from mice. *CALR* expression and mutations are associated with megakaryocytic cells. Murine Ba/F3 and 32D cells are not characterized by megakaryocytic features associated with *CALR*-mutated MPNs. It is well known that distinct types of cells act differently; therefore, the obtained results can also vary because of the chosen cellular model’s additional mutational spectrum, as in the case of the MARIMO cell line where a 61 bp deletion is present. Here, we intended to replicate the cellular model, which is more like the *CALR*-mutated cells found in humans, so we focused on a cell line with megakaryocytic features, i.e., UT-7 cells in which common *CALR* mutations were initiated. Only a few studies have analyzed *CALR’s* role of *CALR* in MPN pathogenesis using circulating CD34^+^ cells or cells cultured from blood or bone marrow samples of patients with MPN [38,44,45,46,47]. In agreement with our results, Abba et al. [44] found constitutive phosphorylation of STAT5 in circulating CD34^+^ cells harboring *CALR* mutations compared to cells with non-mutated *CALR*. Unfortunately, the limited amount of research related to patient cell samples, as well as the distinct types of performed experiments and their targets, leads to the inability to perform an in-depth comparison between our study and others. We have to accept that the ‘clinical’ cell model is desirable for analyzing MPNs’ biological processes and would allow us to find the differences in cell signaling system activation between patient-based and genome-modified cells.

It has been reported that *CALR* mutants are associated with MPL, which leads to the activation of JAK/STAT, PI3K/Akt/mTOR, and MAPK [51,55,56,59,60,86]. We must admit that we performed experiments with cells lacking *MPL* expression, i.e., genome-edited UT-7 cells harboring *CALR* 52 bp deletion or 5 bp insertion. Thus, it can be hypothesized that the JAK/STAT signaling pathway in *CALR*-mutated cells may also be activated in an MPL-independent manner.

The activated PI3K/Akt/mTOR signaling pathway plays one of the main roles in MPN pathogenesis [60]. However, there is little data that shows the impact of mutated *CALR* on PI3K/Akt/mTOR signaling cascade activation. It was found that *CALR* mutants induced phosphorylation of JAK2, STAT1, STAT3, and STAT5, but the PI3K/Akt/mTOR and MAPK pathways were weakly activated [51,55] in the Ba/F3 cell line expressing human *MPL*. Meanwhile, *CALR*-mutant 32D cells without exogenous MPL showed increased activation of AKT [36]. Here, in our study, the association of mutated *CALR* with the PI3K/Akt/mTOR system was assessed, and this complex analysis showed that cells with a *CALR* 52 bp deletion and *CALR* 5 bp insertion are characterized by activated PI3K/Akt/mTOR. *CALR*-mutated cells were more sensitive to RAD001 inhibitors compared to cells with wild-type *CALR*. Moreover, gene expression analysis revealed increased expression of the *RPS6KB1* gene in both *CALR*-mutated cell lines. Although after Western blot analysis, the S6K1 protein levels were not detected in all tested cell lines, significantly increased levels of phosphorylated 4E-BP1 were observed in *CALR* Ins5 cells. These observations indicate that the PI3K/Akt/mTOR signaling pathway might be a new potential target for the treatment of *CALR*-mutated MPN. It is important to note that these are the very first results indicating PI3K/Akt/mTOR signaling activation through mutant *CALR* (together with the lack of MPL expression), so the need arises for further research regarding the association between CALR and the PI3K/Akt/mTOR pathway.

It is necessary to identify alternative cell signaling pathways that might be involved in the pathogenesis of MPN mutant clones, and this could be targeted alone or in combination with JAK/STAT for improved therapeutic benefit [67,87,88]. Several studies have shown that the Hedgehog signaling pathway plays important roles in normal hemopoiesis and the pathogenesis of myeloid malignancies [74,78,87,88]. An increase in the expression of Hh target genes, including *GLI1* and *PTCH1*, was observed by quantitative PCR in granulocytes isolated from MPN patients compared to normal controls. The Hh pathway activity was also found in a murine bone marrow model of PMF [74]. It has been suggested that components of the Hh pathway cooperate with TGFβ, p53, and mTOR-related genes to produce the biological phenotype of PMF [76]. The most recent study was performed using the *C. elegans* model that naturally lacks *JAK2* and *MPL* expression [77]. *CALR* 52 bp deletion and 5 bp insertion were introduced into nematodes using CRISPR/Cas9 methodology. It was found that *CALR*-mutant worms were characterized by the aberrant expression of signal transducers and receptors in the Hh signaling pathway. It was concluded that most of the target genes were downregulated in the *C. elegans* model with *CALR* mutation; mutant calreticulin alone, without JAK2/MPL intervention, can affect transcriptional alterations in Hh signaling. Contrary to these results, a preclinical model showed that granulocytes from MPN patients had elevated Hh target gene expression compared to controls [74], but the *CALR* mutational status was not mentioned in this study. Moreover, Hedgehog ligand inhibitors fully targeted the Hh pathway in murine *JAK2* p.V617F positive cells [89]. Here, we revealed that *CALR*-mutated cells were more sensitive to the Hedgehog inhibitor, HPI-1, compared to the UT-7 cell line used as a control. However, the expression of the *PTCH1* gene, which encodes the target of the Hh signaling pathway, was not detected, and subsequently, the analysis of protein levels was omitted. Therefore, it is important to note that due to inconclusive data, there is a need to expand current experiments analyzing mutant *CALR* and Hedgehog association.

In our previous study, we found that SET-2 cells expressing *JAK2* p. V617F mutation showed increased sensitivity toward the tested agents compared to control UT-7 cells [84]. CYT387 inhibited the proliferation of SET-2 cells with the lowest IC50 compared to RAD001 and HPI-1 inhibitors. In this study, gene expression and Western blot analysis confirmed JAK/STAT signaling pathway activation in SET-2 cells. Elevated STAT1 and STAT5 gene expression and higher STAT1 and STAT5 protein levels were observed. Our findings acknowledged other study results where the rate of *JAK2* mutation-positive cell proliferation, gene expression, and protein levels after JAK/STAT inhibitors exposure, were analyzed [3,64,90,91,92]. Moreover, the results of our study confirm those of previous reports, suggesting that cells harbor *JAK2* p. The V617F mutation is sensitive to mTOR inhibitors such as RAD001 [3,64]. The IC50 value was greater compared to other studies, but this could be due to different cell cultivation conditions, types of plastics, and assays used for cell viability determination [64]. Nevertheless, the activation of PI3K/Akt/mTOR in SET-2 cells was confirmed by gene expression and protein level analyses, in which elevated *RPS6KB1* gene expression and significant phosphorylation of 4E-BP1 were observed. To date, preclinical data on the potential role of the Hh signaling pathway in MPN with mutated *JAK2* are limited. Only one study showed that the expression of Hh signaling molecules increased up to 100-fold in granulocytes isolated from patients with MPNs [74]. Therefore, cell-based in vitro studies on the effects of Hh pathway inhibitors on MPN cells are warranted. Our previous study was the first to address the antiproliferative effect of HPI-1 on cells carrying *JAK2* mutations. We found that SET-2 cells were significantly more sensitive to HPI-1 inhibitors compared to the wild-type *JAK2* cell line [84]. However, no *PTCH1* gene, which encodes one of the Hh signaling targets, was expressed in SET-2 cells, and subsequently, analysis of protein levels was omitted.

Finally, in our study, we conducted a mutated calreticulin functional analysis. It is known that oxidative stress induces the pathological accumulation of ROS that damages membrane lipids, proteins, and DNA [40,41,81,82,92,93,94]. As mentioned before, several studies have shown the importance of ROS in MPN pathogenesis; e.g., ROS has one of the major roles in disease progression, as ROS is a mediator of *JAK2* p.V617F-induced DNA damage [79,80,81]. However, calreticulin plays multiple roles in a variety of cell processes, including responses to oxidative stress [95,96,97]. It has been determined that *CALR* overexpression increased cell sensitivity to H_2_O_2_-induced cytotoxicity and played a critical role in oxidative stress-induced apoptosis [97]. Only a few studies have analyzed the effect of mutated *CALR* on oxidative stress in vitro, where *CALR* mutants were expressed in K562 and CD34^+^ cells [40,41]. Although K562 cells lack *MPL* expression, these cells express the *BCL-ABL1*, and this is the main limitation of using K562 cells as an MPN disease model. Nevertheless, naturally, in MPNs, *CALR* mutations do not occur together with *BCR-ABL1*. Therefore, more accurate conclusions about the association between mutated *CALR* and oxidative stress would be made if *BCR-ABL1* signaling was blocked (e.g., with Imatinib) in the K562 cell line. However, K562 cells expressing mutated *CALR* showed increased ROS levels and increased levels of DNA damage upon oxidative stress exposure to Melittin and Miltirone. In addition, *CALR* mutants were characterized by a decreased ability to reduce intracellular ROS levels and repair oxidative DNA damage compared to *CALR* wild-type K562 cells. Genovese et al. data described how *JAK2* and *CALR* mutations affect oxidative response in CD34^+^ cells from patients PMF patients and healthy donors [41]. It was determined that ROS increased significantly in both *JAK2*- and *CALR*-mutated cells compared to CD34 + cells from healthy donors. It is important to note that *CALR*-mutated cells showed higher ROS levels than *JAK2*-mutated cells. Moreover, *CALR*-mutated CD34^+^ cells were unable to reduce ROS levels, while other tested cells efficiently counteracted intracellular ROS accumulation. The results also showed an increase in apoptosis and a greater oxidative stress effect on DNA damage in *CALR*-mutated cells [41]. Here, our study results agreed with the previously mentioned results and showed an association between increased levels of intracellular ROS and mutated *CALR*. Our data indicate higher levels of ROS, apoptosis, and DNA damage compared to the results of Salati et al. and Genovese et al. [40,41]. Moreover, we analyzed cell cycle distribution after H_2_O_2_-induced oxidative stress, and cell cycle arrest at the G2/M phase in *CALR* Del52 and *CALR* Ins5 cells was determined. This has not been analyzed in previous studies, and our data suggest that the accumulation of G2/M-*CALR*-mutated cells indicates that oxidative stress-induced DNA damage is difficult to repair. The differences in ROS levels, DNA damage, and apoptosis induction may be because of the different cells used in the analysis, as well as the agents used for oxidative stress induction. Salati et al. experiments were carried out using K562 cells carrying *CALR* 52 bp deletion or 5 bp insertion as well as the *BCR-ABL1* variant [40]. Furthermore, the mutation, whether *CALR* 52 bp deletion or *CALR* 5 bp insertion was identified in PMF patients, was not specified in the Genovese et al. study [41]. Therefore, it is not clear which *CALR* mutation has a higher impact on ROS accumulation, apoptosis induction, and oxidative stress-induced DNA damage. In our study, we tried to replicate an MPN cellular model with UT-7 cells after the introduction of *CALR* 52 bp deletion and *CALR* 5 bp insertion. UT-7 cells are not characterized by the expression of activating *BCR-ABL1* signaling or typical mutations that are found to significantly initiate cell proliferation and the pathophysiological state. In the mentioned studies, two main agents were used as oxidative stress inducers, namely H_2_O_2_ [97] and Melittin [40,41]. Here, we used a well-known and approved oxidative stress inducer H_2_O_2,_ while Melittin is quite a new agent inducing oxidative stress in human suspension cells [98]. Despite differences between the studies that were conducted, the tendency for *CALR* 52 bp deletion and 5 bp insertion to impair native calreticulin function is still present. To sum up, *CALR*-mutated cells are more sensitive to oxidative stress, which leads to increased DNA damage.

Our study provides a broad description of the molecular mechanisms that are activated in cells carrying *CALR* mutations. However, this study has certain limitations that need to be acknowledged. First, analysis related to the presence of a double mutation when both *JAK2* and *CALR* mutations occur could be carried out. Although there are a few cases of MPNs with double mutations, it is still not clear how this affects the pathogenesis of these diseases. Moreover, as with most studies of this type, more signaling pathways with corresponding molecules involved in pathogenic processes can always be investigated. Therefore, it would be useful to investigate the activity of other signaling pathways (e.g., MAPK, Aurora A) in cells with *CALR* 52 bp deletion and 5 bp insertion. In addition, because increased activity of JAK/STAT and PI3K/Akt/mTOR signaling pathways was found in *CALR*-mutated cells, it would be possible to study the response of cells with mutated *CALR* to drugs (e.g., ruxolitinib and anagrelide) that are currently used in the treatment of MPN (here, we used specific inhibitors that are still only at the preclinical stage but effective against targets in the tested signaling pathways). Despite the limitations mentioned, the discussed findings provide a more detailed understanding of the complex biology of mutated calreticulin, which might be used to create new molecularly targeted therapeutic strategies for MPNs in the future.

## 4. Materials and Methods

### 4.1. Cell Culture

**Cell lines.** Suspension cell lines, SET-2 and UT-7, were obtained from The German Collection of Microorganisms and Cell Cultures (DSMZ, Braunschweig, Germany). DSMZ carried out the mentioned cell line authentication. The *JAK2* p.V617F mutation and wild-type *CALR* are characteristics of the SET-2 cell line. SET-2 cells were grown in RPMI-1640 medium (Gibco, Gaithersburg, MD, USA) supplemented with 20% fetal bovine serum (FBS), 100 U/mL penicillin, 100 µg/mL streptomycin, and 2 mM L-glutamine (Gibco, Gaithersburg, MD, USA). The UT-7 cell line exhibited wild-type *CALR*, *JAK2*, and *EPOR*. In addition, UT-7 cells lack *MPL* expression [99,100]. The UT-7 cell line was cultured in α-MEM medium (Gibco, Gaithersburg, MD, USA) supplemented with 20% FBS, 100 U/mL penicillin, 100 µg/mL streptomycin, 2 mM L-glutamine (Gibco, Gaithersburg, MD, USA), and recombinant human granulocyte macrophage-colony stimulating factor (Sigma-Aldrich, St. Louis, MO, USA) at a final concentration of 5 ng/mL in the prepared medium.

**DNA constructs and *CALR* gene targeting.** CRISPR/Cas9 system was utilized to initiate *CALR* 52 bp deletion and 5 bp insertion in the UT-7 cell line. Megakaryocytes play a central role in the pathogenesis of ET and PMF; therefore, we focused on a cell line with megakaryocytic features, i.e., UT-7 cells. The UT-7 cell line is a good model for examining MPL-independent molecular pathways behind the effect of the *CALR* 52 bp deletion and *CALR* 5 bp insertion [99,100].

The *CALR* gene sequence was obtained from the GenBank sequence collection of the National Center for Biotechnology Information (National Biosciences, Inc., Bethesda, MD, USA). The CRISPR specially developed design tools, *CRISPR* Design from Zhang Lab (https://www.zlab.bio/) and SnapGene (GSL Biotech LLC, Boston, MA, USA), were used to generate single guide RNA (sgRNA) patterns targeting distinct portions of the *CALR* gene.

For the establishment of sgRNA, two targets in the *CALR* gene were chosen, i.e., for each common *CALR* gene mutation, and, subsequently, nucleotide sequences with the sgRNA generating region were produced. Briefly, the nucleotide sequences with the sgRNA encoding pattern were produced, annealed, phosphorylated, and cloned into a human codon-optimized *S. pyogenous* Cas9-sgRNA vector (pSpCas9(BB)-2A-Puro (pX459) V2.0 (#62988) plasmid (Addgene, Teddington, UK). Constructs were introduced by chemical transformation into competent *E. coli* DH5α for cloning purposes using a selectable marker of Ampicillin. Finally, plasmid construct amplification in *E. coli* DH5α was accomplished, and plasmid DNA was extracted and validated.

In parallel, single-stranded oligodeoxynucleotide donor templates were prepared for each *CALR* gene mutation. Single-stranded oligodeoxynucleotide donor templates were used for the endogenous cellular repair pathway, homology-directed repair (HDR), which was employed to repair CRISPR/Cas9-generated double-stranded DNA breaks and subsequently led to precise alterations at the specified genomic location.

Electroporation was applied for UT-7 cell line co-transfection of the generated CRISPR/Cas9 plasmid and HDR donor template. The UT-7 cell line was cultured in T75 flasks (TPP; Trasadingen, Switzerland) and pelleted by centrifugation at 300× *g* for 5 min. It is known that inhibiting non-homologous end joining (NHEJ) or promoting HDR, either genetically or pharmacologically, leads to increased knock-in efficiency. Here, we chose cell cycle synchronization at the S and G2/M phases that are necessary for HDR, using serum-depleted medium because the serum starvation-re-feeding method had no impact on UT-7 cell proliferation. Therefore, UT-7 cells were serum-deprived for 24 h and restimulated to enter the S and G2/M phases by re-feeding cells with 20% serum for 24 h before electroporation. The cell cycle distribution was analyzed using a Muse Cell Cycle Kit (protocol is presented in Section 4.7) at the end of each period. Further, the cells were pelleted and counted using a Neubauer hemocytometer (Weber Scientific, Hamilton, OH, USA) under an optical microscope. UT-7 cells were suspended in laboratory-made electroporation medium at a concentration of 16.875 × 10^6^ cells/mL. Next, a volume of 50 µL (0.54 × 10^6^ cells) was transferred between stainless steel plate electrodes separated by a 2 mm gap. The final concentration of the pRJ1-del52 and pRJ3-ins5 plasmid constructs was 0.295 µg/mL, whereas the *CALR* mutation-specific HDR template concentration was 4 µM. A BTX T820 electroporator (Harvard Apparatus) (Artisan Technology Group, Champaign, IL, USA) was used for pulsing and changing the voltage and pulse duration. A square wave 1 HV (i.e., high voltage) pulse, 1400 V/cm pulse strength, and 250 µs pulse duration were applied. The electroporation regimen was carried out at room temperature. After pulsing, the cells were transferred into 24-well (TPP; Trasadingen, Switzerland) plates and left for 20 min for recovery. After the recovery period, UT-7 cells were seeded into a 24-well plate, which was supplemented with a culture medium of up to 450 µL. Afterward, the plates were placed in a cell culture incubator for 48 h.

Thereafter, the transfection medium was replaced with a selective medium containing 1 µg/mL puromycin (Gibco, Gaithersburg, MD, USA), and a single clone isolation method was used to select each cell that carried a *CALR* 52 bp deletion and *CALR* 5 bp insertion. A fresh puromycin-containing medium was changed every other day, and stable cell colonies with initiated specific *CALR* mutations were selected and grown to the amounts needed for further applications.

A PCR-based amplicon length differentiation assay was applied to confirm the presence of the *CALR* 52 bp deletion and *CALR* 5 bp insertion in the genome of a putative genome-modified cell line [101].

All tested cell lines were grown in a standard cell culture incubator maintained at 37 °C, 100% relative humidity, and 5% CO_2_. Cell morphology and number were routinely checked using an Olympus CK40 phase contrast microscope (Olympus Corporation, Tokyo, Japan).

### 4.2. Cell Viability Assays

**Signaling system inhibitors.** The mTOR signaling inhibitor RAD001 (Everolimus) (targeting TORC1) was purchased from Alfa Aesar (Alfa Aesar, Haverhill, MA, USA). CYT387 (Momelotinib), an ATP-competitive JAK1/JAK2 inhibitor (Abcam, Cambridge, UK), was also used in this study. Hedgehog signaling pathway inhibitor-1 (HPI-1) (active against Gli1/Gli2) was purchased from Sigma-Aldrich (Sigma-Aldrich, St. Louis, MO, USA). All the mentioned inhibitors were diluted at room temperature with 100% dimethylsulfoxide (DMSO) (Sigma-Aldrich, St. Louis, MO, USA,) to a concentration of 10 mM. The prepared inhibitor solutions were stored at −20 °C and used only once in the experiments.

**AlamarBlue test.** Cell proliferation was analyzed by the colorimetric alamarBlue cell viability assay (Thermo Fisher Scientific, Waltham, MA, USA) using a Tecan Sunrise^TM^ plate reader (Tecan, Manedorf, Switzerland). 24 h before the experiment, 5000 cells were seeded into a 96-well plate (Greiner CELSTAR^®^, Kremsmünster, Germany). Cells were exposed to specific inhibitors (or an appropriate amount of the inhibitor solvent DMSO) for 24, 48, and 72 h. Following the inhibitor incubation, 10% of the well’s volume of alamarBlue reagent was added, and the plates were then placed in the incubator, which was maintained at 37 °C with 5% CO_2_ and 100% relative humidity. Absorbance was measured at wavelengths of 550 nm and 620 nm, and subsequently, cell viability was assessed using the formula provided in the manufacturer’s protocol. Three independent experiments were performed with five replicates per experiment. Cell viability was normalized to that of the control cells.

**Trypan exclusion assay.** A trypan dye solution (Gibco, Gaithersburg, MD, USA) was utilized to assess the viability of the cells. 2 × 10^5^ cells were seeded into 35 mm diameter Petri plates (TPP; Trasadingen, Switzerland) 24 h before the experiment. The cells were treated with suitable doses of inhibitors of particular signaling systems (or inhibitor solvent DMSO) at 24, 48, and 72 h. Following incubation, the cells were collected by centrifugation (300 × *g* for 5 min) and 90 µL of 0.4% trypan blue dye was added to 10 µL of the cell suspension. Using an Olympus CK40 microscope (Olympus Corporation, Tokyo, Japan) and a Neubauer hemocytometer chamber, the viability of the cells was evaluated. Three separate experiments were carried out. For every experiment, three replicates were performed. Cell viability was normalized to control cells.

The concentration at which 50% inhibition (IC50) of cell proliferation occurred was calculated using Excel add-in ED50V10.

### 4.3. RNA Extraction and Reverse Transcription-Quantitative PCR

The JAK/STAT, PI3K/Akt/mTOR, and Hedgehog signaling components *EIF4EBP1*, *RPS6KB1*, *STAT1*, *STAT5A*, and *PTCH1* genes were included in the gene expression analysis. The expression of target genes in SET-2, *CALR* Del52, and *CALR* Ins5 cells was evaluated using reverse transcription-quantitative PCR (RT-qPCR). The UT-7 cell line was used as a control. RNA isolation was performed with the RNeasy Mini Kit (Qiagen, Hilden, Germany) using 1 × 10^6^ cells, following the manufacturer’s recommendations. RNA quantity was assessed using a Thermo Scientific^TM^ Multiskan Sky^TM^ Microplate Spectrophotometer (Thermo Fisher Scientific, Waltham, MA, USA). RNA integrity was analyzed by agarose gel electrophoresis before cDNA synthesis. RNA samples were stored at −80 °C until use.

Two micrograms of RNA was used for cDNA synthesis using the High-Capacity cDNA Reverse Transcription Kit (Applied Biosystems, Foster City, CA, USA), and gene expression was measured using TaqMan Expression Assays (*EIF4EBP1* Hs00607050_m1, *RPS6KB1* Hs00356367_m1, *STAT1* Hs01013996_m1, *STAT5A* Hs00234181_m1, and *PTCH1* Hs00181117_m1) (Invitrogen, Waltham, MA, USA). According to the manufacturers’ recommendations, RT-qPCR was performed using a 7500 Fast Real-time PCR system (Applied Biosystems, Foster City, CA, USA). Utilizing the comparative Ct method, expression data were evaluated after being normalized to the expression levels of the *ACTB* (Invitrogen, Waltham, MA, USA) gene.

### 4.4. Protein Extraction and Western Blot Analysis

1 × 10^6^ cells/well were seeded into 6-well (TPP; Trasadingen, Switzerland) plates for protein level analysis. The cells were lysed using RIPA lysis buffer (Abcam, Cambridge, UK) with proteinase (Sigma-Aldrich, St. Louis, MO, USA) and phosphatase (Sigma-Aldrich, St. Louis, MO, USA) inhibitors. After removal of the cell culture medium by centrifugation at 300× *g* for 5 min, the cells were washed twice with cold 1X PBS, collected by centrifugation, and further incubated with 100 µL RIPA buffer for 20 min. After incubation, the cell lysate was centrifuged at 10,000× *g* for 20 min at 4 °C to pellet the cell debris. The supernatant was collected, and protein concentration was measured using the Pierce^TM^ BCA Protein Assay Kit (Thermo Fisher Scientific, Waltham, MA, USA) and Thermo Scientific^TM^ Multiskan Sky^TM^ Microplate Spectrophotometer (Thermo Fisher Scientific, Waltham, MA, USA) at 570 nm according to the manufacturer’s recommendations. Protein samples were stored at −20 °C until use.

After denaturing the samples at +70 °C, 40 µg of protein was loaded onto NuPage^TM^ 4–12% Bis-Tris Plus Gels (Invitrogen, Waltham, MA, USA) for vertical protein electrophoresis. After electrophoresis, the proteins were transferred to a PVDF membrane (Invitrolon^TM^ PVDF/Filter Paper Sandwich, Invitrogen, Waltham, MA, USA) using a semi-wet transfer unit Mini Blot module (Invitrogen, Waltham, MA, USA). Further, the membrane was blocked with a blocking buffer and incubated overnight at +4 °C with a primary antibody against phospho-STAT1 Tyr701 (#33-3400, Invitrogen, Waltham, MA, USA), STAT1 (#SC-464, Santa Cruz Biotechnology, Dallas, TX, USA), phospho-STAT5 Tyr694 (#71-6900, Invitrogen, Waltham, MA, USA), STAT5 (#44-368G, Invitrogen, Waltham, MA, USA), phospho-4E-BP1 Ser65 (#MA-14948, Invitrogen, Waltham, MA, USA), 4E-BP1 (#9644, Cell signaling Technology, Danvers, MA, USA), phospho-S6K1 Thr389 (#9205, Cell signaling Technology, Danvers, MA, USA), phospho-S6K1 Thr389 (#9234S, Cell signaling Technology, Danvers, MA, USA) and S6K1 (#2708S, Cell signaling Technology, Danvers, MA, USA), and β-actin (#AM-4302, Invitrogen, Waltham, MA, USA). After washing with 1X Pierce^TM^ TBS Tween20 buffer (Thermo Fisher Scientific, Waltham, MA, USA), the blots were incubated with the relevant Anti-Rabbit HRP-conjugated, Anti-Mouse HRP-conjugated, or Anti-Mouse Alkaline-Phosphatase-conjugated secondary antibody solution (Invitrogen, Waltham, MA, USA). Before detection, the blots were washed with 1X Pierce^TM^ TBS Tween20 buffer and distilled water. The proteins were detected with Super Signal^TM^ West Pico PLUS Chemiluminescent Substrate, SuperSignal^TM^ West Atto Ultimate Sensitivity Substrate (Thermo Fisher Scientific, Waltham, MA, USA), or Novex™ AP Chemiluminescent Substrate (CDPStar™) (Invitrogen, Waltham, MA, USA) for the detection of horseradish peroxidase (HRP) and alkaline phosphatase (AP), respectively, according to the manufacturer’s recommendations. Chemiluminescent images were visualized using the ChemiDoc^TM^ XRS+ system (Bio-Rad Laboratories, Hercules, CA, USA) and analyzed with ImageLab 6.0.1 Software (Bio-Rad Laboratories, Hercules, CA, USA). β-actin protein was used as a loading control for the normalization of the target protein expression level.

### 4.5. Detection of Intracellular Reactive Oxygen Species

Hydrogen peroxide (H_2_O_2_) was added to the cell culture to induce oxidative stress. Intracellular reactive oxygen species (ROS) were evaluated using a flow cytometry-based method. 24 h before the experiment, 1 × 10^6^ cells were seeded in a 6-well plate. For 24 h, the cells were subjected to an H_2_O_2_ solution (concentration 400 µM and 0 µM). The cells subjected to 500 µM H_2_O_2_ solution served as a positive control to calibrate the instrument parameters. The Muse Oxidative Stress Kit (Merck Millipore, Burlington, MA, USA) was used to assess cell populations under oxidative stress, following the manufacturer’s instructions. Subsequently, cell analysis was performed, and the signal was detected using a Muse Cell Analyzer (Merck Millipore, Burlington, MA, USA).

The ability of the cells to reduce oxidative stress after H_2_O_2_ exposure was also evaluated. Cells exposed to H_2_O_2_ were transferred to a medium without hydrogen peroxide and incubated for 24 h. ROS levels were determined according to a previously described protocol. The software package Muse 1.4 was utilized to ascertain the percentage of cells experiencing oxidative stress.

### 4.6. Apoptosis Level Analysis

24 h before the experiment, 2 × 10^5^ cells were seeded in a 6-well plate. Cells were exposed to H_2_O_2_ (concentration 0 and 400 µM) solution for 24 h. After exposure, cell apoptosis analysis was performed using the Muse Annexin V and Dead Cell Kit (Merck Millipore, Burlington, MA, USA) according to the manufacturer’s recommended protocol. Cell analysis was performed, and the signal was detected using a Muse Cell Analyzer (Merck Millipore, Burlington, MA, USA). The obtained data were analyzed using the Muse 1.4 software package.

### 4.7. Cell Cycle Assay

The changes in cell cycle distribution were assessed with the Muse Cell Analyzer using the Muse Cell Cycle Kit (Merck Millipore, Burlington, MA, USA). Cells were seeded in a 6-well plate 24 h before the experiment (1 × 10^6^ cells per well). The cells were exposed to H_2_O_2_ (concentration 0 and 400 µM) solution for 24 h. Next, the analysis of the cell cycle was performed according to the manufacturer’s protocol. The signal was detected with a cell analyzer, and the results were analyzed using the Muse 1.4 software package.

### 4.8. DNA Damage Measurement

The Muse Multi-Color DNA Damage Kit (Merck Millipore, Burlington, MA, USA) was used for DNA damage assessment. 24 h before the experiment, 1.5 × 10^6^ cells were seeded in a 6-well plate. Cells were exposed to H_2_O_2_ (concentration 0 and 400 µM) solution for 24 h. Cells exposed to 10 µM etoposide were used as a positive DNA damage control. After exposure, analysis of DNA damage was performed according to the manufacturer’s protocol. The signal was detected using the Muse Cell Analyzer, and the results were analyzed using the Muse 1.4 software package.

The ability of cells to repair DNA damage caused by oxidative stress was also assessed. The cells that were exposed to H_2_O_2_ were transferred to a medium without the addition of hydrogen peroxide and incubated for an additional 24 h, and DNA lesions were assessed according to a previously described protocol.

### 4.9. Statistical Analysis

IBM SPSS Statistics 22.0 (IBM Corp., Armonk, NY, USA) software was used to perform statistical analysis. Results were determined to be normally distributed using the Shapiro–Wilk test for normality. Data were analyzed using an independent sample *t*-test and one-way analysis of variance (ANOVA), followed by a post-hoc Tukey’s multiple comparison test when appropriate. All experiments were repeated at least three times, with three to five replicates. All data are shown as the means with standard deviation (S.D.), while the *p* < 0.05 value was considered statistically significant.

## 5. Conclusions

Our research provides novel evidence on *CALR*’s association with JAK/STAT, PI3K/Akt/mTOR, and Hedgehog signaling pathways that are important in MPN pathogenesis. Our data indicate that the JAK/STAT and PI3K/Akt/mTOR signaling pathways are activated in cells carrying the *CALR* 52 bp deletion and *CALR* 5 bp insertion. To unravel the MPL-independent mechanisms underlying the effect of *CALR* mutations, we performed experiments in a cell culture model lacking MPL expression. This research is unique because most studies have analyzed mutant calreticulin when *MPL* expression is present. We determined that the activation of the mentioned signaling pathways does not necessarily depend on the interaction between CALR and MPL. We also showed that *CALR* gene mutations impair physiological calreticulin function, leading to reduced responses to oxidative stress and DNA damage. Functional analysis revealed that the accumulation of G2/M-*CALR*-mutated cells indicates that oxidative stress-induced DNA damage is difficult to repair. The mentioned cell cycle delay has not been shown in other studies analyzing mutated calreticulin. Taken together, this study contributes to a deeper understanding of the specific molecular mechanisms underlying *CALR*-mutated myeloproliferative neoplasms.

## Data Availability

The data are contained in the article or Supplementary Material. Further inquiries can be directed to the corresponding author.

## Supplementary Materials

The following supporting information can be downloaded at https://www.mdpi.com/article/10.3390/ijms25189873/s1.
