# Supplementary material for: The Role of Mutated Calreticulin in the Pathogenesis of BCR-ABL1-Negative Myeloproliferative Neoplasms
Source: Int J Mol Sci. 2024 Sep 12;25(18):9873. doi: 10.3390/ijms25189873 (PMC11432199; doi:10.3390/ijms25189873)
Supplement: Supplementary file 1 [file ijms-25-09873-s001.zip › ijms-3185274-supplementary.pdf]

Table S1. IC_{50} values of tested inhibitors against *CALR* Del52 and *CALR* Ins5 cells.

	RAD001 mean IC50 (SD)	CYT387 mean IC50 (SD)	HPI-1 mean IC50 (SD)	P value	95% CI of the difference
***CALR* Del52 cells**					
alamarBlue assay					
24 h					
RAD001 *vs* CYT387	43.97 (12.62)	43.73 (3.93)		0.999	19.24 to 19.27
RAD001 *vs* HPI-1	43.97 (12.62)		40.38 (2.11)	0.843	15.89 to 23.07
CYT387 *vs* HPI-1		43.73 (3.93)	40.38 (2.11)	0.861	16.13 to 22.83
48 h					
RAD001 *vs* CYT387	42.32 (13)	2.71 (0.02)		0.001	22.42 to 60.55
RAD001 *vs* HPI-1	42.32 (13)		27.66 (1.8)	0.122	4.40 to 33.73
CYT387 *vs* HPI-1		2.71 (0.02)	27.66 (1.8)	0.012	7.76 to 45.89
72 h					
RAD001 *vs* CYT387	18.58 (0.32)	0.39 (0.19)		0.0001	17.46 to 19.02
RAD001 *vs* HPI-1	18.58 (0.32)		18.36 (0.31)	0.693	0.57 to 1.0
CYT387 *vs* HPI-1		0.39 (0.19)	18.36 (0.31)	0.0001	17.24 to 18.81
Trypan blue exclusion assay					
24 h					
RAD001 *vs* CYT387	43.21 (5.35)	37.41 (6.19)		0.261	5.27 to 19.41
RAD001 *vs* HPI-1	43.21 (5.35)		42.22 (4.81)	0.694	8.96 to 15.71
CYT387 *vs* HPI-1		37.41 (6.19)	42.22 (4.81)	0.650	8.64 to 16.03
48 h					
RAD001 *vs* CYT387	41.05 (6.32)	0.84 (0.05)		0.0001	32.29 to 47.33
RAD001 *vs* HPI-1	41.05 (6.32)		26.93 (0.80)	0.02	8.27 to 23.30
CYT387 *vs* HPI-1		0.84 (0.05)	26.93 (0.80)	0.0001	16.51 to 31.55
72 h					
RAD001 *vs* CYT387	19.32 (0.53)	0.34 (0.2)		0.0001	17.97 to 20.05
RAD001 *vs* HPI-1	19.32 (0.53)		17.44 (0.54)	0.002	0.97 to 3.05
CYT387 *vs* HPI-1		0.34 (0.2)	17.44 (0.54)	0.0001	15.96 to 18.04
***CALR* Ins5 cells**					
alamarBlue assay					
24 h					
RAD001 *vs* CYT387	49.68 (9.34)	47.53 (5.12)		0.924	15.10 to 19.40
RAD001 *vs* HPI-1	49.68 (9.34)		31.52 (5.35)	0.051	0.91 to 35.40
CYT387 *vs* HPI-1		47.53 (5.12)	31.52 (5.35)	0.066	1.24 to 33.25
48 h					
RAD001 *vs* CYT387	25.92 (0.73)	0.80 (0.17)		0.0001	22.79 to 27.47
RAD001 *vs* HPI-1	25.92 (0.73)		21.72 (1.43)	0.004	1.87 to 6.55
CYT387 *vs* HPI-1		0.80 (0.17)	21.72 (1.43)	0.0001	18.58 to 23.26
72 h					
RAD001 *vs* CYT387	22.31 (1.05)	0.61 (0.42)		0.0001	19.85 to 23.55
RAD001 *vs* HPI-1	22.31 (1.05)		19.61 (0.59)	0.010	0.86 to 4.55
CYT387 *vs* HPI-1		0.61 (0.42)	19.61 (0.59)	0.0001	17.15 to 20.85
Trypan blue exclusion assay					
24 h					
RAD001 *vs* CYT387	42.76 (1.95)	46.24 (15.40)		0.927	42.29 to 35.32
RAD001 *vs* HPI-1	42.76 (1.95)		35.56 (4.20)	0.741	31.60 to 46.01
CYT387 *vs* HPI-1		46.24 (15.40)	35.56 (4.20)	0.553	28.12 to 49.49
48 h					
RAD001 *vs* CYT387	27.01 (0.23)	0.47 (0.43)		0.0001	22.33 to 30.75
RAD001 *vs* HPI-1	27.01 (0.23)		22.94 (1.68)	0.055	0.15 to 8.27
CYT387 *vs* HPI-1		0.47 (0.43)	22.94 (1.68)	0.0001	18.27 to 26.69
72 h					
RAD001 *vs* CYT387	21.01 (0.85)	0.32 (0.27)		0.0001	18.34 to 23.04
RAD001 *vs* HPI-1	21.01 (0.85)		20.09 (0.39)	0.358	1.43 to 3.28
CYT387 *vs* HPI-1		0.32 (0.27)	20.09 (0.39)	0.0001	22.13 to -17.41
